# Prosomeric Hypothalamic Distribution of Tyrosine Hydroxylase Positive Cells in Adolescent Rats

**DOI:** 10.3389/fnana.2022.868345

**Published:** 2022-05-06

**Authors:** María G. Bilbao, Daniel Garrigos, Marta Martinez-Morga, Angel Toval, Yevheniy Kutsenko, Rosario Bautista, Alberto Barreda, Bruno Ribeiro Do-Couto, Luis Puelles, José Luis Ferran

**Affiliations:** ^1^Consejo Nacional de Investigaciones Científicas y Técnicas (CONICET), Buenos Aires, Argentina; ^2^Facultad de Ciencias Veterinarias, Universidad Nacional de La Pampa, General Pico, Argentina; ^3^Department of Human Anatomy and Psychobiology, School of Medicine, University of Murcia, Murcia, Spain; ^4^Institute of Biomedical Research of Murcia – IMIB, Virgen de la Arrixaca University Hospital, Murcia, Spain; ^5^PROFITH “PROmoting FITness and Health Through Physical Activity” Research Group, Department of Physical Education and Sports, Faculty of Sport Sciences, University of Granada, Granada, Spain; ^6^Department of Human Anatomy and Psychobiology, Faculty of Psychology, University of Murcia, Murcia, Spain

**Keywords:** POMC, hypothalamic dopamine, terminal hypothalamus, peduncular hypothalamus, acroterminal hypothalamus, arcuate nucleus, paraventricular nucleus, A13 group

## Abstract

Most of the studies on neurochemical mapping, connectivity, and physiology in the hypothalamic region were carried out in rats and under the columnar morphologic paradigm. According to the columnar model, the entire hypothalamic region lies ventrally within the diencephalon, which includes preoptic, anterior, tuberal, and mamillary anteroposterior regions, and sometimes identifying dorsal, intermediate, and ventral hypothalamic partitions. This model is weak in providing little or no experimentally corroborated causal explanation of such subdivisions. In contrast, the modern prosomeric model uses different axial assumptions based on the parallel courses of the brain floor, alar-basal boundary, and brain roof (all causally explained). This model also postulates that the hypothalamus and telencephalon jointly form the secondary prosencephalon, separately from and rostral to the diencephalon proper. The hypothalamus is divided into two neuromeric (transverse) parts called peduncular and terminal hypothalamus (PHy and THy). The classic anteroposterior (AP) divisions of the columnar hypothalamus are rather seen as dorsoventral subdivisions of the hypothalamic alar and basal plates. In this study, we offered a prosomeric immunohistochemical mapping in the rat of hypothalamic cells expressing tyrosine hydroxylase (TH), which is the enzyme that catalyzes the conversion of L-tyrosine to levodopa (L-DOPA) and a precursor of dopamine. This mapping was also combined with markers for diverse hypothalamic nuclei [agouti-related peptide (*Agrp*), arginine vasopressin (*Avp*), cocaine and amphetamine-regulated transcript (*Cart*), corticotropin releasing Hormone (*Crh*), melanin concentrating hormone (*Mch*), neuropeptide Y (*Npy*), oxytocin/neurophysin I (*Oxt*), proopiomelanocortin (*Pomc*), somatostatin (*Sst*), tyrosine hidroxilase (*Th*), and thyrotropin releasing hormone (*Trh*)]. TH-positive cells are particularly abundant within the periventricular stratum of the paraventricular and subparaventricular alar domains. In the tuberal region, most labeled cells are found in the acroterminal arcuate nucleus and in the terminal periventricular stratum. The dorsal retrotuberal region (PHy) contains the A13 cell group of TH-positive cells. In addition, some TH cells appear in the perimamillary and retromamillary regions. The prosomeric model proved useful for determining the precise location of TH-positive cells relative to possible origins of morphogenetic signals, thus aiding potential causal explanation of position-related specification of this hypothalamic cell type.

## Introduction

The catecholamines dopamine, norepinephrine, and epinephrine are neurotransmitters that, among other functions, intervene in motor output, reward, learning, memory processing, and endocrine modulation (Björklund and Dunnett, 2007). The hypothalamic dopaminergic system, which includes several intrinsic dopaminergic cell populations, apparently modulates the responses of several hypothalamic nuclei. One well-known example is the tuberal arcuate nucleus (Arc), whose intrinsic dopaminergic neurons modulate their own activity, as well as the functions of the alar paraventricular (PA) neurons involved in energy homeostasis (Zhang and van den Pol, 2016). Most works on rodents that identified dopaminergic neurons in the hypothalamus focused on the description of tyrosine hydroxylase (TH) and/or dopamine (DA) positive neurons within the columnar model (e.g., Swanson et al., 1981; Chan-Palay et al., 1984; Ruggiero et al., 1984; Van den Pol et al., 1984; Tillet, 1994). This enzyme catalyzes the conversion of L-tyrosine to levodopa (L-DOPA), a precursor to DA, the latter being itself a precursor of norepinephrine and epinephrine. These studies observed that various populations of hypothalamic cells contain TH, the first enzyme involved in the catecholamine synthesis pathway, and essentially lack expression of dopamine-β-hydroxylase (DBH; found elsewhere in noradrenergic neurons), L-aromatic acid decarboxylase (AADC), and phenylethanolamine-N-methyltransferase (PNMT; typical of adrenergic neurons) (Swanson and Hartman, 1975; Swanson et al., 1981; Chan-Palay et al., 1984; Van den Pol et al., 1984; Jaeger, 1986; Ugrumov et al., 1989; Foster, 1994, 1998). Ross et al. (1984) only detected PNMT in some hypothalamic cell bodies after blockage of axonal flow by administration of colchicine, but immunoreactions usually show positive fibers and terminals (Foster, 1994). Foster (1994, 1998) and Ugrumov (1994) compared systematically TH with AADC neurons (Jaeger, 1986) in the developing rat hypothalamus and observed somewhat retarded overlapping expression of AADC in some TH-positive hypothalamic cell groups (e.g., A12, A13, and A14/A15), but could not confirm co-expression of both markers. In summary, according to these studies, most hypothalamic cells that express TH in rodents are presumably dopaminergic (Swanson and Hartman, 1975; Swanson et al., 1981; Chan-Palay et al., 1984; Van den Pol et al., 1984; Ugrumov et al., 1989; Björklund and Dunnett, 2007).

Cells containing catecholamines (CA) in the hypothalamus were first described in rats and several other species using the Falck-Hillarp histofluorescence reaction; this approach was followed by immunofluorescence and inmunohistochemical techniques (Dahlström and Fuxe, 1965; Björklund and Nobin, 1973; Fuxe et al., 1978; Swanson et al., 1981; Chan-Palay et al., 1984; Van den Pol et al., 1984). The diverse descriptions of catecholaminergic neurons in the diencephalic region failed in general to ascribe distinct CA cell groups to the individual columns of the columnar model but did recognize as hypothalamic neuronal groups identified alphanumerically as A11, A12, A13, A14, and A15 (Figure 15A; Dahlström and Fuxe, 1964; Björklund and Nobin, 1973; Chan-Palay et al., 1984; Ruggiero et al., 1984; Van den Pol et al., 1984; Ugrumov et al., 1989).

Only a few studies analyzed the hypothalamic TH/*Th* expressing cells in rodents using the neuromeric model (e.g., Puelles and Medina, 1994; Puelles and Verney, 1998; Marín et al., 2005; Puelles et al., 2012a,b). Puelles and Medina (1994) and Puelles and Verney (1998) used a primitive simpler version of the prosomeric model, whose hypothalamic region was later substantially revised by Puelles et al. (2012a). The latter authors classified A11 as diencephalic (plurineuromeric, as suggested already by Puelles and Medina, 1994) and placed A13 within a caudal basal subregion of peduncular hypothalamus (PHy). This notion was raised previously by Tillet (1994); p.211, who proposed a double hypothalamic and zona incerta (prethalamic) location of A13, possibly due to confusion with the rostral part of A11. A12 was ascribed to basal terminal hypothalamus (Thy), divided into dorsal tuberal and ventral tuberal subpopulations separated by the VM nucleus, plus an acroterminal arcuate population. The A14 group was identified as occupying the alar domains of both PHy and THy, while the A15 group was ascribed to the telencephalic preoptic area.

The adolescent period of Sprague-Dawley (SD) rats is usually defined between P30 and P60 and registers extensive neurobiological changes in large regions of the brain (Spear, 2000; Caballero and Tseng, 2016). In addition, this period is characterized by a larger gain in weight and adipose tissue content compared to the young adult stage (Kutsenko et al., 2021). Accurate characterization of the distribution of TH-positive cells in the hypothalamic region during the adolescent period is important for experimental designs aiming to determine their action during modulation of motor and metabolic responses in this period of life (Caballero and Tseng, 2016; Toval et al., 2017, 2020, 2021; Kutsenko et al., 2021). The aim of the present study was to map more precisely the distribution of TH-positive neurons in specific nuclei or strata of the hypothalamic region in adolescent rats following the updated area map provided by the prosomeric model (Puelles et al., 2012a; Ferran et al., 2015a; Puelles and Rubenstein, 2015). We analyzed in detail the distribution of TH-positive cells in hypothalamic alar and basal domains and subdomains of SD rats during late adolescence (P45 to P60). Immunohistochemistry for TH was performed on floating agarose sections and partly combined with *in situ* hybridization for [agouti-related peptide (Agrp), arginine vasopressin (Avp), cocaine and amphetamine-regulated transcript (Cart), corticotropin releasing Hormone (Crh), melanin concentrating hormone (Mch), neuropeptide Y (Npy), oxytocin/neurophysin I (Oxt), proopiomelanocortin (Pomc), somatostatin (Sst), tyrosine hidroxilase (Th), and thyrotropin releasing hormone (Trh)] mRNA to identify relationships with specific hypothalamic peptidergic cell populations. The study shows a broader distribution of TH-positive cells than was observed in earlier works, emphasizing specific hypothalamic nuclei and strata. These results will be useful for future experiments exploring the causal mechanisms involved in the specification of TH-positive neurons, and also in functional studies that consider the intrinsic dopaminergic sources that may modulate the activity of diverse hypothalamic populations or their pathological changes.

**FIGURE 1:**
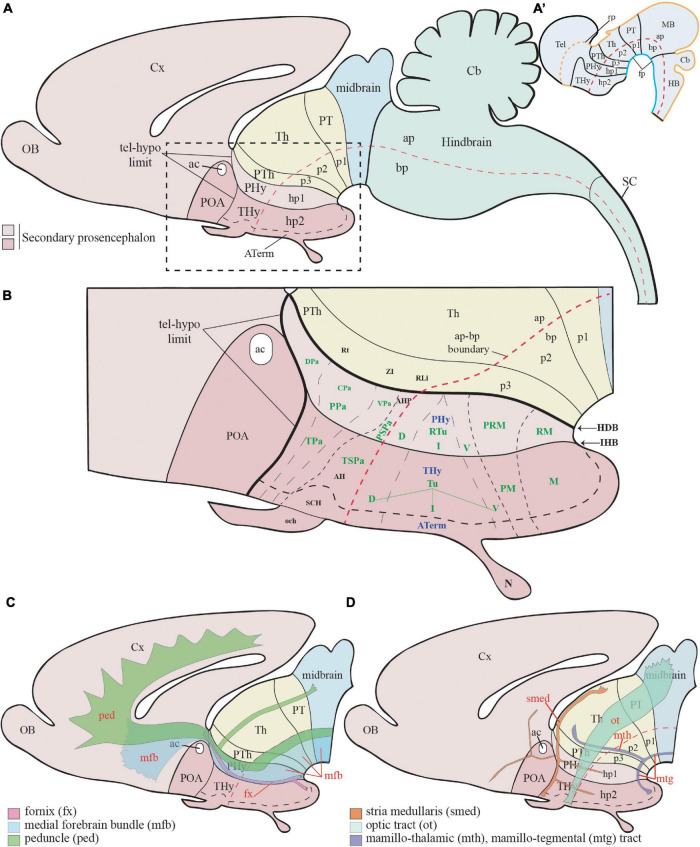
Schematic representation of the main hypothalamic partitions according to the prosomeric model. **(A)** According to the updated prosomeric model, the forebrain includes the midbrain (blue), the diencephalon (yellow), and the secondary prosencephalon or hypothalamo-telencephalic region (light and dark pink). The hypothalamic forebrain region is a ventral part of two rostral hypothalamo-telencephalic prosomeres (hp1 and hp2) of the secondary prosencephalon. The hypothalamic region includes alar (ap) and basal (bp) plate derivatives (separated by a red dash-line) but excludes de alar preoptic area (POA), which belongs to the telencephalon (see Abbreviations for the names of major forebrain regions below). **(A′)** Schema showing the early neural tube at stages of the initial development of the prosomeric partitions. **(B)** Schema of the terminal (dark pink; THy) and peduncular (light pink; PHy) anteroposterior hypothalamic parts and their analogous main dorsoventral partitions according to the prosomeric model. Red dashes, alar-basal boundary; continuous black lines orthogonal to red dashes = transverse interneuromeric borders (AP pattern); IHB, intrahypothalamic (hp1/hp2) border. HDB, hypothalamo-diencephalic border. Short black dashes = primary DV alar and basal divisions; long black dashes = secondary DV subdivisions (DV pattern). **(C)** Schematic representation of the main dorsoventral tracts that run through the PHy (ped, mfb, fx). The fx and mfb limit with the intrahypothalamic boundary (IHB); the ped only limits with the HDB. **(D)** Schema of the main longitudinal tracts that run through THy and PHy (smed, ot, and mtg). Only the smed tract shows some TH-positive fibers. For abbreviations see the list. ac, anterior commissure; AH, anterior hypothalamic nucleus; AHP, anterior hypothalamic nucleus peduncular; ap, alar plate; ATerm, acroterminal region; bp, basal plate; Cb, cerebellum; CPa, central paraventricular nucleus; cVPA, caudal ventral paraventricular nucleus; Cx, cortex; D, dorsal tuberal/retrotuberal región; DPa, dorsal paraventricular nucleus; fp, floor plate; fx, fórnix; HDB, hypothalamo-diencephalic boundary; hp1, hypothalamic prosomere 1; hp2, hypothalamic prosomere 2; I, intermediate tuberal/retrotuberal region; IHB, intrahypothalamic boundary; M, mamillary region; MB, midbrain; mfb, medial forebrain bundle; mtg, mamillo-tegmental tract; mth, mamillo-thalamic tract; N, neurohypophysis; och, optic chiasma; p1, prosomere 1; p2, prosomere 2; p3, prosomere 3; ped, peduncle; PHy, peduncular hypothalamus; PM, perimamillary region; POA, preoptic area; PPa, peduncular prosomere paraventricular area; PRM, periretromamillary region; PSPa, peduncular subparaventricular area; PT, pretectum; PTh, prethalamus; RLi, rostral liminar area; RM, retromamillary region; Rt, reticular nucleus; RTu, retrotuberal area; SC, spinal cord; SCH, suprachiasmatic nucleus; smed, stria medullaris; Th, thalamus; Thy, terminal hypothalamus; TPa, terminal paraventricular area; TSPa, terminal subparaventricular area; Tu, tuberal hypothalamic área; V, ventral tuberal/retrotuberal region; VPa, ventral paraventricular nucleus; ZI, zona incerta.

A detailed area map of the hypothalamic wall (e.g., Figures 1A,B) presupposes a radial ventriculo-pial dimension. The radial mantle strata are defined in the prosomeric model as *periventricular, medial, lateral*, and *superficial strata* (Puelles et al., 2012a). The classic *medial* and *lateral* strata (Crosby and Woodburne, 1940) can be understood as inner and outer parts of a standard intermediate stratum. The location of the classical A12 to A15 TH-positive groups will be described within the alar and basal partitions of the two hypothalamic prosomeric portions (PHy and THy), considering, as well as the radial distribution of the immunoreactive neurons.

The neuromeric mapping (anteroposterior (AP) pattern) also clarifies the topologic relationships of CA cell groups in the hypothalamus with the corresponding longitudinal zones (distance vector (DV) pattern), providing an underpinning for causal mechanistic and comparative analysis of the developmental or phylogenetic origins/derivations of the individual cell groups (see early efforts along this line in the book “Phylogeny and Development of Catecholamine Systems in the CNS of Vertebrates,” Smeets and Reiner, 1994). In some cases, this perspective occasionally suggests reasons for splitting previously lumped complexes, particularly in context with modern knowledge about molecularly differently defined progenitor domains.

## Materials and Methods

The experimental procedures were approved by the Animal Research Ethics Committee (CEEA) of the University of Murcia (Authorization Number: REGA ES300305440012) and were carried out in accordance with the guidelines on the use of animals for scientific purposes in Spain (RD 53/2013, Law 32/2007) and the European Union (86/609/EEC). The study followed the FORCED guidelines according to the housing and animal conditions (Garrigos et al., 2021).

### Animals

Male SD rats, between 45 and 60 postnatal days, were provided by the animal facilities of the University of Murcia. SD rats were weighed and housed under the same conditions in standard size cages (50 cm × 35 cm × 35 cm with a 2–3 cm dry cork), shared by three animals, and replaced every 4–5 days. A 12:12 h light-dark cycle was set up and the brains were obtained during the active phase of the rats (dark cycle). The rooms were kept at a temperature of 22 to 25°C with a relative humidity of 45 to 60%. The animals had *ad libitum* access to a standard chow diet (ENVIGO, diet 2014, United States) and filtered water.

### Brain Processing

Sprague-Dawley brains were obtained and processed following the protocols of Ferran et al. (2015b,c). The brains were perfused with a saline solution followed by phosphate-buffered 4% paraformaldehyde (0.1 M PB; pH 7.4). Once extracted, the brains were maintained at 4°C for 24 h. Some brains were then washed in PBS, followed by 15 and 30% sucrose solutions in 0.1 M phosphate-buffed saline (PBS) solution (pH 7.4) and cut with a sliding microtome (Micron HM430, Thermo Scientific, United States) into sagittal, horizontal, and transversal sections (50 μm thick). Sections were collected as parallel or consecutive series on SuperFrost Plus slides (Menzel-Gläser, Braunschweig, Germany) and processed for hybridization and/or immunohistochemistry. A few other brains were washed with PBS and embedded in 4% agarose (low electroendosmosis-EEO agarose; catalog No. 8008; Pronadisa, Spain) to obtain vibratome sections (100 μm thickness) and processed as floating sections for immunohistochemistry (Ferran et al., 2015b,c).

### RT-PCR and Cloning

*Agrp, Cart, Mch, Npy, Oxt, Pomc, Sst, Th*, and *Trh* cDNA fragments were obtained by RT-PCR and cloned into TA vectors to later synthesize the RNA probes. Fresh brain tissues from postnatal rats were sheared by 1 cycle of 20 s at 6500 RPM in 2 ml tubes (CK14) using the Precellys Evolution system (Bertin Technologies, France). The RNA was then extracted from the samples using the NZY total RNA isolation kit (Nzytech, MB13402, Portugal) and treated with DNaseI (Invitrogen, Cat. 18068-015, United States). Conversion to cDNA was performed with Superscript III reverse transcriptase (Invitrogen, Cat. 18080-044, Spain) and oligo dT anchored primers. The cDNA was used as a template for PCR using Taq polymerase (Promega, Cat. M8305, Spain) and specific primers (Supplementary Material 1). The resulting PCR products were then cloned into the pGEM-T Easy Vector (Promega, Cat. A1360, Spain) and sequenced by ACTI (University of Murcia, Spain).

### *In situ* Hybridization

Brain sections obtained from sliding microtome were collected in SuperFrost slides and processed according to *in situ* in cryosections as described in Ferran et al. (2015c). Digoxygenin-11-UTP (Roche, Lewes, United Kingdom) was used for the synthesis of labeled RNA sense and antisense riboprobes from cDNA samples of *Agrp, Avp, Cart, Crh, Mch, Npy, Oxt, Pomc, Sst, Th*, and *Trh* rat genes. Probe sequence information is provided in Supplementary Material 2. Linear cDNA templates were obtained by amplifying cloned fragments by PCR (Ferran et al., 2015b).

### Immunohistochemistry

A detailed version of the immunohistochemical reaction has been described in previous work (Ferran et al., 2009, 2015c). Highlighting key steps, tissues were initially exposed to 0.3% hydrogen peroxide to inactivate endogenous peroxidases. The primary antibody was incubated overnight at 4°C (TH [NB300-109], Novusbio, 1:200 dilution, Bio-Techne R&D Systems, Spain). After the washes, the sections were incubated with secondary antibody for 2 h (Biotinylated anti-rabbit (H+L), Vector Laboratories, United States, 1:1 dilution). Next, a streptavidin-peroxidase complex (Vectastatin-ABC kit; Vector Laboratories, United States; 0.001% dilution) was applied for 1 h at room temperature. Finally, the sections were washed, and the peroxidase activity was developed with 0.03% 3,30-diaminobenzidine (Sigma, St. Louis, MO, United States), plus 0.003% hydrogen peroxidase. The specificity of the antibody was demonstrated by western blotting (see below) and by performing control experiments that omitted the primary antibody without observing residual immunostaining.

### Western Blot

Fresh rat postnatal brain tissue from the mesencephalic-diencephalic region containing the tegmental ventral area (VTA) was sheared by 1 cycle of 20 s at 6500 RPM in 2 ml tubes (CK14) using the Precellys Evolution system (Bertin Technologies, France). The sheared tissue was resuspended in RIPA lysis buffer (Millipore, United States) and using protease inhibitors (Pierce Biotechnology Inc., United States) and PMSF 1M (Abcam, United Kingdom) for 30 min at 4°C. Protein concentration was initially determined by the Bradford protein system (Sigma-Aldrich, Germany), these were then separated in 10% SDS-polyacrylamide gel (PAGE-SDS, United Kingdom) to be transferred to a nitrocellulose membrane (Whatman, United Kingdom). As standard size in western-blot protein electrophoresis, PageRuler™ Prestained Protein Ladder (Thermo Scientific, United States) was used. After completion of the transfer, the protein bands were visualized by staining the nitrocellulose membranes with Ponceau S solution (Sigma-Aldrich, Germany). Blots were then incubated overnight at 4°C with rabbit antibodies against TH (1:200, Novusbio, Bio-Techne R&D Systems, Spain). The secondary antibody (anti-rabbit IgG (H+L), Vector Laboratories, United States) was used at 1:10.000 for HRP peroxidase (Thermo Fisher, United States). The enhanced chemiluminescence (ECL) Western blot detection system (Amersham Biosciences, United Kingdom) and LuminataTM Forte (Millipore Corporation, United States) was used to determine the immureativity of the TH antibody, using the ImageQuant LAS 500 Gel Documentation System (GE Healthcare, United States). The molecular weight obtained for TH was approximately 60 kDa (Supplementary Material 3).

### Imaging

Processed *in situ* and immunohistochemistry sections were digitalized with a ScanScope CS digital slide scanner (Aperio Technologies, Vista, CA, United States). Size, contrast, brightness, and focus in the images were adjusted by applying Adobe Photoshop CS3. Figures were produced using Adobe Illustrator CS2 (Adobe Systems Inc., San Jose, CA, United States).

## Results

We will describe our findings in DV order, dealing first with the alar hypothalamic domains (PA and subparaventricular (SPa) areas, with a note on the neighboring preoptic region), and then continuing with the underlying basal domains (tuberal, perimamillary, and mamillary areas and corresponding retrotuberal components).

### Tyrosine Hydroxylase-Positive Fiber Tracts

Some tracts known to course along the dorsoventral or anteroposterior axes in the hypothalamic territory are visible in our material and thus help to recognize the hypothalamic structure. The main dorsoventral tracts connecting the telencephalon with the hypothalamus and other territories–the medial and lateral forebrain bundles–pass through the peduncular hypothalamus or PHy (Puelles et al., 2012a; Puelles and Rubenstein, 2015). The TH-negative *fornix tract* (fx) coming from the hippocampal region passes behind the anterior commissure (anterior wall of the interventricular foramen) and follows a strictly dorsoventral course in the PHy region just behind the intrahypothalamic boundary (radially its position approximates the limit between medial and lateral hypothalamic strata). The fornix ends contralaterally after giving collaterals to the mammillary body and thereafter crossing the retromamillary decussation (fx; Figures 1B,C, 3B and Supplementary Figure 6C). Dispersed throughout the peduncular lateral hypothalamic stratum there is the *medial forebrain bundle* (mfb); this complex tract contains many ascending or descending components, including the TH-positive axons from the ventral tegmental area (VTA; mesocortical pathway, mc) and substantia nigra pars compacta (SNC; nigrostriatal pathway, ns) (ns/mc; Figures 1B,C, 4–6, 8). The mfb tract is only present in the PHy, and thus helps to identify the latter’s curved rostral intrahypothalamic boundary with THy (ns/mc; Figure 4), as occurs likewise with the fornix. Superficial to the mfb and running dorsoventrally through the superficial TH-negative PHy stratum there is the *cerebral peduncle* (ped) or *lateral forebrain bundle*. This contains among other (basal ganglia) components corticothalamic, corticopontine, corticoreticular, corticonuclear, and corticospinal fibers (ped; Figures 1C, 6, 8).

Regarding the landmark longitudinal tracts, we can identify in our material the stria medullaris, the optic tract, and the mamillotegmental tract, as well as its collateral mamillothalamic tract. The *stria medullaris tract* (smed) arises mainly from a variety of septocommissural, diagonal, preoptic, and hypothalamic neurons (all possibly migrated from the prethalamic eminence; Alonso et al., 2020). Their axons fasciculate together in the dorsal PHy next to the prethalamic eminence, laterally to the fornix tract after the latter passes behind the anterior commissure. The compacted smed then runs anteroposteriorly through the whole prethalamic eminence and thereafter continues caudalwards next to the chorioidal taenia thalami until it incorporates into the habenular commissure, projecting all the while on the lateral and medial habenula (Figures 1B,D, 4A,B, 8; Puelles et al., 2012a). Several opportunistic TH-positive mfb fibers, either from the ns/mc tracts or from intrinsic hypothalamic TH neurons, send collaterals or swerve into the initial smed approximately at the preopto-hypothalamic boundary, thus causing the smed to appear TH-positive in our material (smed; black arrowheads; Figures 4A,B, 8). The TH-negative optic tract (ot) can be identified from suprachiasmatic levels (the acroterminal SPa alar region) running successively through the THy and PHy alar plate, to follow also longitudinally through the prethalamic, thalamic, pretectal, and mesencephalic alar plate to find its multiple targets (Figures 1B,D, 4, 5D, 6A, 7A,B, 11A; Supplementary Figures 8I–K; Puelles et al., 2012a; Puelles, 2022). The TH-negative mamillotegmental tract (mtg) is a longitudinal tract arising from the rostrobasal mamillary region (THy); it runs caudalwards across the retromamillary, diencephalic, midbrain, and isthmic tegmentum, and then ends primarily at the dorsal and ventral tegmental nuclei of rhombomere 1 (Figures 1B,D, 3A, 13, 14; Puelles et al., 2012a). The mtg produces collaterally the TH-negative mamillothalamic tract (mth) which runs ventrodorsally caudally to the zona limitans intrathalamica into the anterior nuclear complex of the thalamus (Figures 1B,D, 3A, 6B, 13, 14; Puelles et al., 2012a).

### Tyrosine Hydroxylase-Positive Cell Groups in the Hypothalamic Alar Plate

This region is constituted by two dorsoventrally arranged progenitor domains and adult subregions, the PA and SPa areas (Puelles et al., 2012a); both Pa and SPa stretch across PHy and THy (Figure 1B). The alar Pa domain limits dorsally with the telencephalic territory and ventrally with the SPa alar domain; caudally, both limit with the diencephalic prethalamus (Figure 1B); the SPa limits ventrally with the alar-basal boundary (Figure 1B). The PA hypothalamic nucleus that develops within Pa is subdivided dorsoventrally into *dorsal*, *central*, and *ventral* parts (DPa, CPa, VPa; Figure 1B). We will also distinguish in our description the *periventricular* stratum (pe), with cells adjacent to the ependym, from the conventional thicker *medial hypothalamic* stratum (mh), occupied by the Pa nuclear complex, found deep to the *lateral* and *superficial hypothalamic strata* (Puelles et al., 2012a; Ferran et al., 2015a; Puelles and Rubenstein, 2015; Díaz and Puelles, 2020). The SPa area is best developed within THy, where it forms the suprachiasmatic nucleus (held to lie within ATerm) as well as the anterior hypothalamic nucleus (SCH; AH; Figure 1B), whereas the reduced posterior part of the anterior hypothalamic nucleus (AHP) characterizes SPa within PHy (AHP; Figure 1B; Puelles et al., 2012a).

#### Periventricular Stratum

**FIGURE 2:**
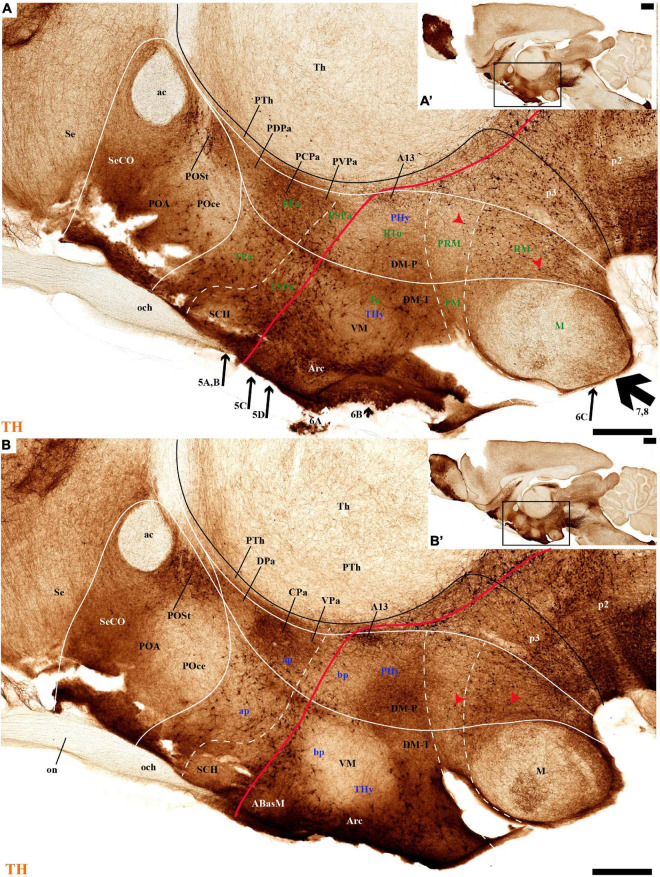
**(A–B′)** TH immunohistochemistry highlighting the preoptic and hypothalamic regions in selected medial sagittal sections of the adolescent rat brain. High and low magnifications of a periventricular section **(A,A′)** and another section close to it **(B,B′)** that distinguish the peduncular (PHy) and terminal (THy) prosomeric partitions of the hypothalamus, including its postulated dorsoventral subdomains. A nearly continuous layer of TH-positive neurons is observed in the periventricular stratum of the terminal/peduncular paraventricular area (TPa/PPa), subparaventricular area (TSPa/PSPa), and a large part of the tuberal and retrotuberal basal plate subdomains (Tu, RTu), excepting the VM and M areas. In addition, some TH-positive neurons are seen in the periventricular stratum of the periretromamillary (PRM) and retromamillary (RM) parts (red arrowhead). In the preoptic region, most TH immunoreactive cells are observed in the strial preoptic region (POSt) with few cells in the central preoptic subregion (POCe) and merely dense neuropil at the septocommissural preoptic subregion (SeCo). The dashed lines identify the boundaries between the hypothalamic dorsoventral subdomains of the alar and basal plate and the red line highlights the alar-basal boundary. A black line indicates the limit between p2 and p3 across the alar and basal plates, whereas another transverse (continuous) or longitudinal (dashes) hypothalamic boundaries appear in white. The section plane of Figures 5–8 are indicated in **(A)**. For abbreviations see the list. Scale bars = 500 μm **(A,B)**; 1000 μm **(A′,B′)**.

The alar periventricular stratum (pe) of TH-positive neurons seems to extend indistinctly across Pa and SPa, as observed in deep sagittal sections (pe; Figures 2A,B), serial horizontal-sections (Figures 5A–D), and serial transversal sections (Figures 7B–D, 8A–C); there are possibly less labeled periventricular elements caudally in PHy –next to prethalamus- while they increase in number rostrally within THy and ATerm (Figures 5A–C); there is a clearcut dorsal limit of this thin alar periventricular sheet with the much less populated preoptic periventricular stratum (POA; Figures 2A,B, 7B–D). In contrast, there is no clear boundary separating the alar periventricular hypothalamic TH neurons from those observed in the basal plate (Figures 2A,B, 7B–D). Cells expressing *Avp, Oxt*, or *Sst* mRNA were described previously in the periventricular stratum of the terminal and peduncular Pa domain (Morales-Delgado et al., 2011; Puelles et al., 2012a). Alar hypothalamic periventricular TH cells coexist indeed with sparse neurons expressing *Avp*, *Trh; Oxt*, or *Sst* mRNA, though the latter are mainly found within PHy and THy, being hardly present within the ATerm domain (Figures 9A–D, 10B,C′, 11B–E″′, 12B–D,H,J,K; Supplementary Figures 4, 5B,C,E,G).

#### Tyrosine Hydroxylase-Cell Groups in Other Strata of the Alar Paraventricular Area

PHy: we describe first the medial stratum of the PHy, whose well-known PA nucleus component (the main component within the whole PA nuclear complex) appears divided in the prosomeric model into dorsal (DPa), central (CPa), and ventral (VPa) subnuclei (Figures 2A,B′, 5A–D, 7C,D, 8A,C, 9B,E, 10C,D′, 11C–E″′, 12D,K and Supplementary Figures 4A,B,E, 5B,C,E,G; see Discussion for comparison with the alternative ‘antero-posterior’ columnar denominations). Our analysis suggests that all three Pa subnuclei display distinguishable radial parts, apart from the previously described general periventricular stratum. The medial hypothalamic stratum at the level of the peduncular PA nucleus is marked by profuse neuropil of TH-positive ramifications, and at some places, it appears subdivided into radial parts (Figure 5).

As regards the medial hypothalamic *dorsal* PA subnucleus (DPa), where some dispersed *Avp, Trh*, and *Sst* neurons are found, the number of peptidergic and TH cells is rather low within the corresponding medial stratum (mainly *Trh* cells; Figure 13), in contrast with the underlying more populated CPa subnucleus. Some TH-positive cells are observed in the superficial DPa layer in the small caudal portion of the SO nucleus that derives from the peduncular prosomere (Figures 10A,B).

Indeed, the medial hypothalamic *central* PA subnucleus (CPa) is richly populated by peptidergic glutamatergic neurons. These can be divided topographically into *inner* and *outer parts* (iC; oC), characteristically described generally as populated, respectively, by parvocellular and magnocellular neurons. However, the existence of mixed cells of both types and other topographic peculiarities elsewhere in CPa leads us to prefer topographic descriptors. The inner part of CPa contains predominantly *Trh*, *Crh*, and *Sst* neurons and some TH cells, whereas the outer part displays a large population of *Avp/Oxt* neurons (iC; oC; Figures 10C,D′, 11C,C′, 12D and Supplementary Figures 4B,D, 5E,F). Only occasionally some TH-positive cells can be observed in the oC partition, which is characterized by *Oxt* mainly in a peripheral ring of cells, whereas *Avp* labels predominantly a core group of cells (Figures 5C, 7C,D, 8A,B, 9E, 10C′, 11C–E″′, 12B,D,H,J and Supplementary Figures 4B–E, 5E,F; Sawchenko and Swanson, 1982, their Figure 1). Caudally to the CPa proper, there appears a thin domain limiting with the thalamus, and tenuously delimited from the CPa, which also shows some peptidergic neurons. This caudal population is conventionally classified within the columnar model as the ‘dorsal Pa nucleus,’ though we interpret it as ‘cCPa,’ that is, as CPa cells lying ‘caudally’ *within the prethalamus* (note the hypothalamus is always separated from the thalamus by the prethalamus, which is reduced morphogenetically to a thin periventricular domain in postnatal animals) (Puelles et al., 2012c,^2021^). The cCPa is likewise characterized by some TH-positive neurons and dispersed cells expressing *Avp, Oxt*, and *Trh* (Figures 5C, 9B,E, 10C–C′, 12B,D,F,H–J and Supplementary Figures 4, 5E,F) (see Discussion and mappings of Gad67 and vGlut2 in Puelles et al., 2012c). This ectopic Pa population, which probably is hypothalamic in origin, must have migrated into prethalamus; the cCPa seems continuous with similar cDPa and cVPa counterparts (Figures 5B,D, 9B,E, 12C,G,K and Supplementary Figures 5B,C,G,H).

The medial hypothalamic *ventral* PA subnucleus (VPa) also can be divided, following the radial dimension, into *inner* and *outer parts* (iV, oV) which roughly resemble in position the iC and oC of CPa; additional more superficial components of VPa lying within the lateral hypothalamic stratum were named *medial* and *lateral lateral* parts (mlV, llV). The iV is characterized by many *Trh*-positive cells mixed with a few cells that express *Avp*, *Oxt*, and only some occasional TH-positive neurons (Figures 5D, 8B,C, 9B,C,E, 11C–E, 12C,G,K and Supplementary Figures 5G,H). The oV contains many cells expressing *Avp* and *Oxt* mRNA, in the absence of *Trh* and TH-positive cells (Figures 11C–E,12C,G,K and Supplementary Figures 5G,H). The mlV is a triangular area lateral to oV, which is stretched lateralwards into a tip that points to the llV; the latter lies interstitial to the deep level of the nigrostriatal tract (Figures 11C,E, 12C,G,K and Supplementary Figures 5G,H). The mlV shows some TH-positive neurons, mixed with few cells expressing *Avp* and *Oxt* mRNA (Figures 5D, 8B,C, 9B,C,E, 11C–E, 12C,G,K and Supplementary Figures 5G,H). The llV is a group of larger peptidergic neurons that express *Avp* which are accompanied occasionally by some TH positive neurons (Figures 9F,F′, 11C–E′, 12C).

**FIGURE 3:**
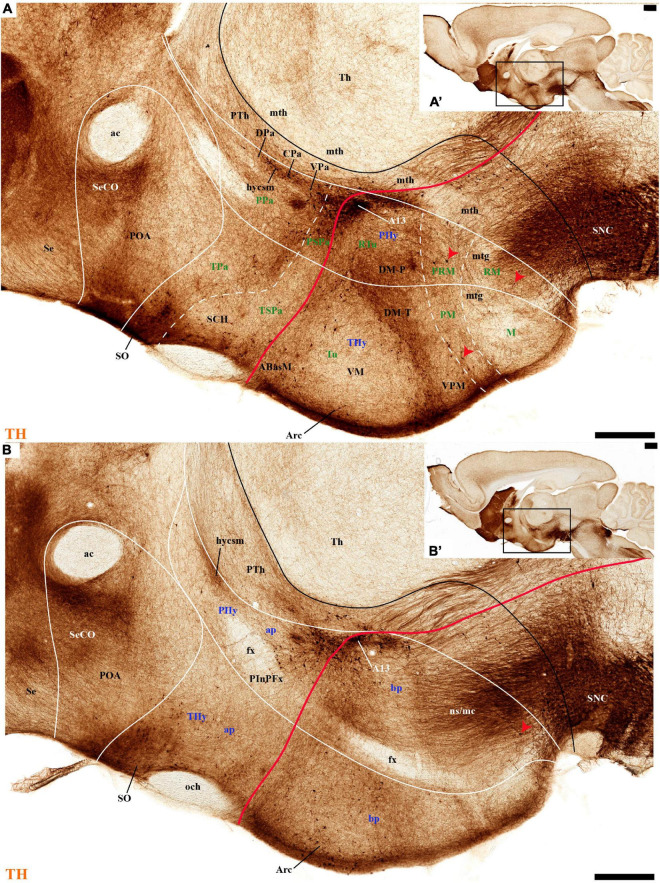
**(A–B′)** TH immunohistochemistry highlighting the preoptic and hypothalamic regions in sagittal sections of the adolescent rat brain. High and low magnification of selected consecutive sagittal sections from the same specimen of Figure 2, distinguishing slightly more lateral levels of the peduncular (PHy) and terminal (THy) prosomeric partitions of the hypothalamic region with their dorsoventral subdomains. Most hypothalamic TH-positive neurons observed occupy the medial stratum (e.g., Pa subnuclei; SPa parts; DM-P, DM-T; ABas; Arc). In addition, few TH-positive neurons are observed in the perimamillary (PM) periretromamillary (PRM) and retromamillary (RM) parts (red arrowhead). The diverse lines follow the code used in Figure 2. For abbreviations see the caption of Figure 1 and Abbreviations List. Scale bars = 500 μm **(A,B)**; 1000 μm **(A′,B′)**.

THy: the Pa nuclear complex derivatives formed within THy (TPa) occupy a much smaller area in dorsoventral extent (Puelles et al., 2012a; their Figure 8.27A); this terminal Pa domain limits dorsally with the preoptic area, ventrally with the SCH and AH nuclei of the SPa area, caudally with the peduncular part of Pa, and rostrally includes the even smaller acroterminal subdomain (Figure 1B). The hypothetic dorsal, central, and ventral TPa parts are not easily distinguished. Apart from the above described TPa pe stratum, some TH neurons characterize likewise its thin medial hypothalamic stratum, delimited by TH-positive general neuropil, which is distinctly thinner in TPa than in PPa (mh; Figures 5C, 7B). In addition, there is a group of dispersed *lateral* TH-positive cells observed selectively outside the outer part of the medial stratum of the terminal PA domain (lTPa; Figures 3B, 5B,C, 7B, 11B and Supplementary Figure 5C). These lateral TH-positive cells can be described instead as belonging to the lateral anterior nucleus of the TPa or the subjacent anterior hypothalamic nucleus of the SPa (LA; Puelles et al., 2012a). These cells typically do not enter the SCH nucleus.

The subpial superficial layer of the TPa domain is characterized by the supraoptic nucleus (SO) which displays many *Avp* and *Oxt* positive neurons, but only shows a few TH-positive cells mixed with them (Figures 3A,B, 5B, 7A, 9C–F, 10A–C, 11A,A′ and Supplementary Figures 4A,A′,C, 8I). Additional superficial TH positive neurons extend from the SO ventralwards, first deep to the chiasma and optic tract, and reach the basal or tuberal suboptic nucleus (TuSbO; name introduced by Puelles et al., 2012a), possibly representing the acroterminal dorsoventral migratory pathway that connects these two formations (Figures 5A,B, 7A, 10A,B, 11A and Supplementary Figure 8I).

#### Tyrosine Hydroxylase-Cell Groups in the Alar Subparaventricular Area

The SPa area is a longitudinal zone located in the ventral part of the alar plate of the terminal and peduncular hypothalamus (Puelles et al., 2012a; Ferran et al., 2015a; Puelles and Rubenstein, 2015; Díaz and Puelles, 2020). The SPa is broad rostrally within ATerm and THy, where the SCH and AH nuclei develop; it narrows caudally within PHy, where the posterior AH (AHP) nucleus is described, which limits with the newly recognized rostral liminar area of the prethalamic alar plate (see Puelles et al., 2021; their Figure 1: SPa; RLi; note this recent interpretation differs from the earlier one of Puelles et al., 2012a). The SPa domain limits ventrally across the alar-basal border with the tuberal and retrotuberal regions of the basal plate (Figure 1B; Puelles et al., 2012a; Ferran et al., 2015a; Puelles and Rubenstein, 2015; Díaz and Puelles, 2020).

Leaving aside the periventricular stratum of SPa, already treated above, we observed some TH cells external to the periventricular stratum that were identified as located in the anterior hypothalamic nucleus (AH) (Figures 5B,C, 7B, 10C′, 11B, 12D). Some cells of the shell of the acroterminal SCH nucleus express *Avp* or *Sst* mRNA, but TH-positive neurons were only occasionally mixed with them. TH-positive cells are detected more frequently around the SCH shell (Figures 2A, 5B, 7A, 9B, 10B, 11A, 12A and Supplementary Figures 5B–D, 6A, 8C,D,I–K). This SCH shell may be derived, perhaps by migration from the Pa area, as is suggested by its expression of *Avp*, which identifies cells derived from the Pa area (Puelles et al., 2012a). The core of the SCH nucleus that lies in the intermediate layer of the acroterminal domain essentially lacks positive TH cells (Figures 3A, 5B, 7A, 9B, 10B, 11A, 12A and Supplementary Figures 5B–D, 6A, 8C,D,I–K). Finally, in the peduncular SPa area, some dispersed TH neurons are observed around the fornix in the *preincertal perifornical nucleus*, which lies where the fornix intersects the SPa AHP (PInPFx; Puelles et al., 2012a; Figures 5D, 8A and Supplementary Figures 9B,C).

### Preoptic Area

**FIGURE 4:**
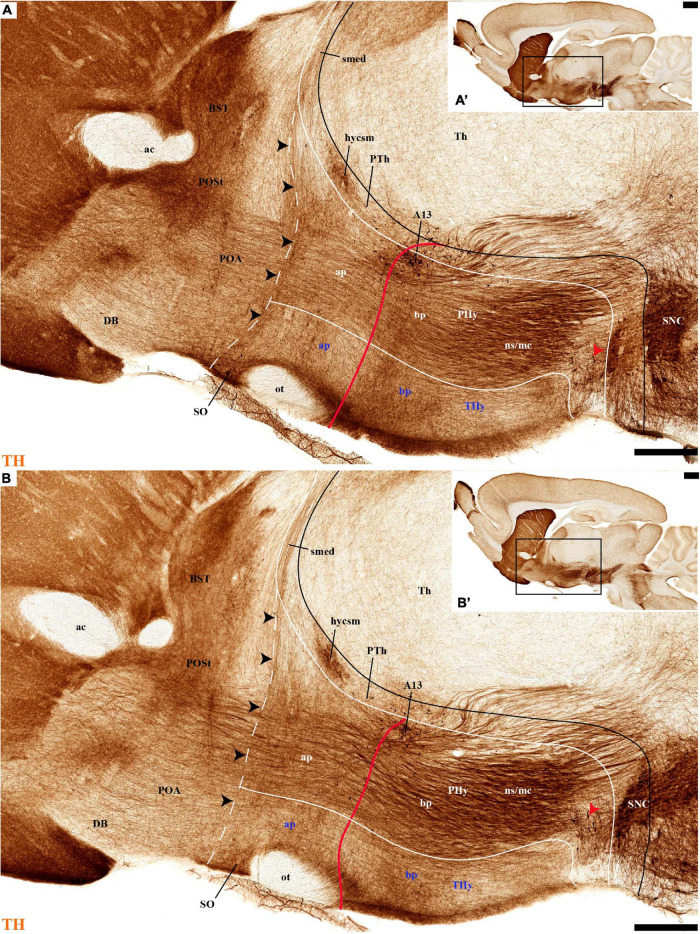
**(A–B′)** TH immunohistochemistry highlighting the preoptic and hypothalamic regions in sagittal sections of the adolescent rat brain. High and low magnification of more lateral selected sagittal sections from the same specimen of Figures 2, 3, at the level of the nigrostriatal tract (ns/nc) distinguishing the peduncular (PHy) and terminal (THy) prosomeric partitions. Most TH-positive neurons are part of the A13 group or SO nucleus. In addition, few TH-positive neurons next to the diencephalic SNc are observed in the lateral retromamillary area (red arrowhead). Dashed lines and black arrowheads identify the boundaries between the preoptic and hypothalamic regions. The diverse lines follow the code used in Figure 2. For abbreviations see the caption of Figure 1 and Abbreviations List. Scale bars = 500 μm **(A,B)**; 1000 μm **(A′,B′)**.

We will place here a few relevant data relative to the preoptic area, which we interpret not as hypothalamic, but as belonging within the hp2 (terminal) prosomere to the telencephalic subpallium territory (this consists of striatal, pallidal, diagonal, and preoptic domains, the preoptic area being the rostralmost of them; Puelles et al., 2012a,^2013^, ^2016^). The preoptic area is limited caudally from the evaginated hp1 telencephalic derivatives by a dorsal transversal extension of the intrahypothalamic boundary. This extends dorsalward into the rostral part of the roof plate, first coursing parallel and just rostrally to the fornix tract (Puelles and Rubenstein, 2015), then bending around the anterior commissure, and reaching the brain surface along with the limit between the septum (part of the evaginated telencephalon) and the preoptic area (unevaginated; Figures 2A,B, 3A,B). The dorsoventral (longitudinal) preopto-hypothalamic border is reported to correlate both with the change from subpallial *Dlx/Arx* preoptic signal versus *Otp/Sim1* hypothalamic PA signal (Shimogori et al., 2010; Puelles et al., 2012a), and with telencephalic *Foxg1* versus hypothalamic *Foxg2* (Hatini et al., 1994). Our data reveal that some fasciculated TH-positive fibers ascending through the medial forebrain bundle diverge caudalwards (longitudinally) into the stria medullaris as they reach this boundary (smed; Figures 4A,B, 8A–D). The preoptic acroterminal domain is formed by the thin and median terminal lamina, which extends between the anterior commissure and the optic chiasma. The median preoptic nucleus is associated with this acroterminal domain; it extends subpially in front of the anterior commissure.

Tyrosine hydroxilase immunoreacted sagittal sections divide the preoptic area into three regions: there is a large *central* region next to the terminal Pa area, whose periventricular stratum is largely devoid of TH neurons (POce). Additionally, there is a *septocommissural* (SeCo) region that represents the preoptic paraseptal transition into the septum (Puelles et al., 2013, 2016); the SeCo shows strong ventricular *Shh* signal and is marked by a profuse TH-positive periventricular neuropil (SeCo; Figures 2A,B, 3A,B, 13A,B). Finally, we distinguish also a separate caudodorsal preoptic region found next to the hp1/hp2 boundary, just ventrally to the anterior commissure; this region may be named the *preopto-strial* nucleus (POSt), since its population apparently contributes to the complexity of the bed nuclei striae terminalis (POSt; Figures 2A,B,4A,B, 8A–D, 9A–E, 11F,H, 12A–E, 13A,B and Supplementary Figure 5A).

The POSt subdivision lies topologically ventral to the anterior commissure and is represented by a dense mass of periventricular TH-immunoreactive neurons that decreases laterally in a triangular shape. The axons from these cells seem to proceed laterocaudally into the supracapsular stria terminalis (Figures 2A,B, 8A–D). The ventral contour of POSt is lined (limited) by *Avp/Oxt* positive cells arranged in a small periventricular group and a larger comma-shaped lateral group forming a partial ventral shell to the POSt population. We named these peptidergic populations *inner and outer preoptic magnocellular nuclei* (iPOMc; oPOMc; Figures 9A,B,D,E, 10B,B″, 11F–H, 12A).

### Basal Plate

The hypothalamic basal plate corresponds to the classic regions of the tuberal, posterior and mamillary hypothalamus.

*THy:* however, according to the prosomeric thesis of dual peduncular and terminal hypothalamic prosomeres, the classic tuberal and mamillary areas strictly belong only to THy (hp2), jointly with a small perimamillary part of the classic posterior hypothalamus (Tu, PM, M; Figure 1B). The Tu is redefined as a *longitudinal* basal THy domain lying under the alar-basal boundary, and divided dorsoventrally into dorsal, intermediate, and ventral subdomains (Tu; DTu, ITu, VTu; Figure 1B; Puelles et al., 2012a; Ferran et al., 2015a; Puelles and Rubenstein, 2015). The Tu area ends rostrally in a distinct tuberal acroterminal subdomain with similar dorsoventral subdivisions and characteristic derivatives described below (ATerm; Figures 1B, 13A, 15A; Puelles et al., 2012a; Puelles and Rubenstein, 2015). Ventrally to the major Tu region there appear two other longitudinal basal plate components, the perimamillary area (PM) and the mamillary area (M) (PM; M; Figure 1B; see molecular delimitations in Puelles et al., 2012a and López-González et al., 2021). These areas also end rostrally in the corresponding ventral part of the basal ATerm (Figure 1B). M contacts ventrally the floorplate of hp2 (Figure 1B).

*PHy:* the PHy displays instead of a smaller, similarly, placed, and subdivided *retrotuberal* region (RTu; RTuD, RTuI, RTuV; Figure 1B), which was not clearly recognized classically. The intrahypothalamic boundary that separates transversally Tu from RTu roughly passes just rostral to the fornix tract (fx; Figures 1C, 3B; Puelles et al., 2012a; Puelles and Rubenstein, 2015). The RTu region contacts caudally the prethalamic tegmentum (basal plate) in the diencephalic prosomere 3 (Figure 1B; Puelles et al., 2012a; Puelles and Rubenstein, 2015). Ventral to RTu we distinguish periretromamillary and retromamillary areas (PRM; RM; Figure 1B); RM was classically known as ‘supramamillary area,’ whereas PRM represents another part of the classic posterior hypothalamus. Note that columnar authors added to posterior hypothalamus other more caudal basal plate regions now ascribed instead to the diencephalic tegmentum (basal p3; basal p2; Figure 1B).

Our description of basal hypothalamic TH cell populations will proceed successively through the Tu/RTu, PM/PRM, and M/RM longitudinal basal domains.

#### TuD/RTuD

**FIGURE 5:**
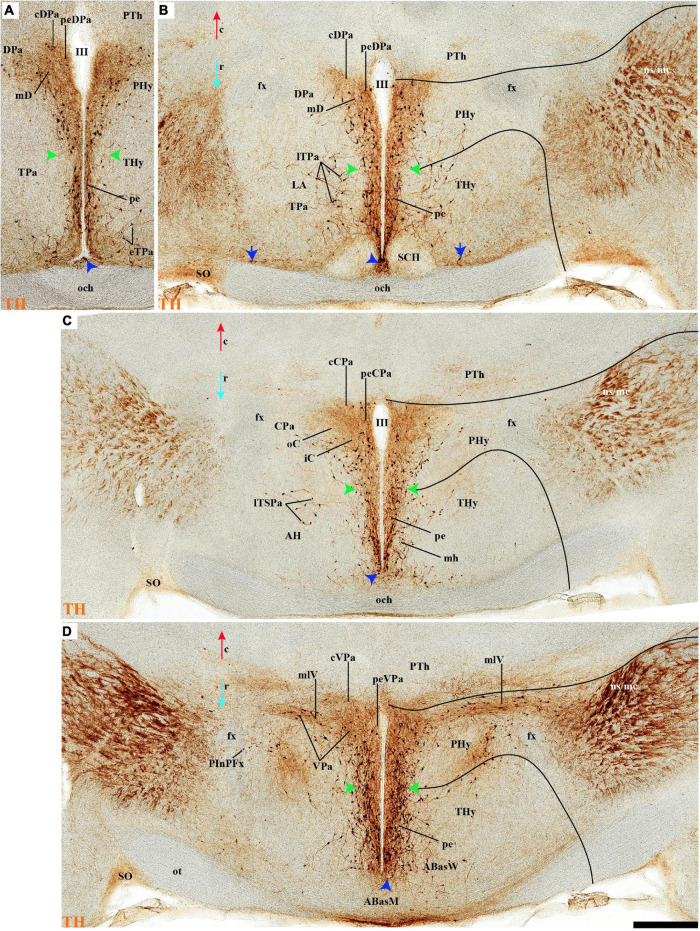
**(A–D)** TH immunohistochemistry highlighting hypothalamic dopaminergic neurons and fibers in selected consecutive horizontal sections of an adolescent rat brain. The series starts at the level of the optic chiasma **(A–D)**. The peduncular (PHy) and terminal (THy) prosomeric limits (green arrowhead and black lines) are identified. TH-positive neurons are observed distinctly in the periventricular stratum (pe), including the pe of the ATerm domain (blue arrowhead) and in some medial strata of specific nuclei. In addition, few TH-positive neurons are observed in the outer (blue arrow) or superficial strata. For abbreviations see the caption of Figure 1 and Abbreviations List. See section planes in Figure 2A. Orientation arrows: red arrow = caudal; blue arrow = rostral. Scale bar = 500 μm.

Abundant TH-positive cells are observed in the pe and adjacent deep stratum of the dorsal tuberal area (TuD), as well as in the correlative dorsal retrotuberal area (RTuD) (Figures 2A,A′, 5D, 7A–D, 11A,B, 13B,B′, 14B,B′ and Supplementary Figures 6A,A′, 9A,B). This longitudinal TuD/RTuD progenitor band produces periventricular, deep, intermediate, and superficial mantle strata; the cited TH cells are found mainly among the periventricular and deep components, also known as the anterobasal and posterobasal nuclei (ABas/PBas; Puelles et al., 2012a; their Figures 8.30 and 8.31). The wing-shaped bilateral longitudinal prolongation of the tuberal ABas nucleus (ABasW) and the retrotuberal PBas nucleus extends over the underlying ventromedial and dorsomedial TuI/RTuI formations, respectively (ABasW; PBas; Figures 7B–D). The intermediate and superficial strata of the TuD domain contain, respectively, the nucleus of the tuber cinereum (TCI) and the tuberal suboptic nucleus (TuSbO; Figures 9C,F, 11A and Supplementary Figures 8I,J; this was known classically as ‘tuberal supraoptic nucleus,’ though its cells clearly lie *under* the optic tract, as was argued by Puelles et al., 2012a when they proposed the alternative, accurate name). The TuSbO contains *Avp* and *Oxt* cells migrated dorsoventrally from the terminal Pa area or the SO nucleus (Díaz et al., 2015). Unlike the SO nucleus, however, which shows sparse TH-positive cells mixed with its *Avp* and *Oxt* cells, TH perikarya are largely absent at the TuSbO nucleus (Figures 9C,F, 11A and Supplementary Figures 8I,J).

Within the corresponding acroterminal territory of TuD we distinguish a median anterobasal area (ABasM), which is known classically as the retrochiasmatic area. The ABasM is intercalated dorsoventrally between the also acroterminal SCH nucleus of the SPa area and the tuberal arcuate nucleus (Arc) of the TuI area (Puelles et al., 2012a; Ferran et al., 2015a; Puelles and Rubenstein, 2015). TH-positive cells are largely absent in ABasM proper, but the local pe stratum shows some scattered immunoreactive TH cells (ABasM; Figures 5D, 7A, 10C,C′, 11A, 12D and Supplementary Figures 4A–B). A few TH-positive cells appear also in the superficial stratum of the acroterminal TuD, possibly related to the TuSbO nucleus (Figures 5C,D and Supplementary Figures 4B, 5E).

**FIGURE 6:**
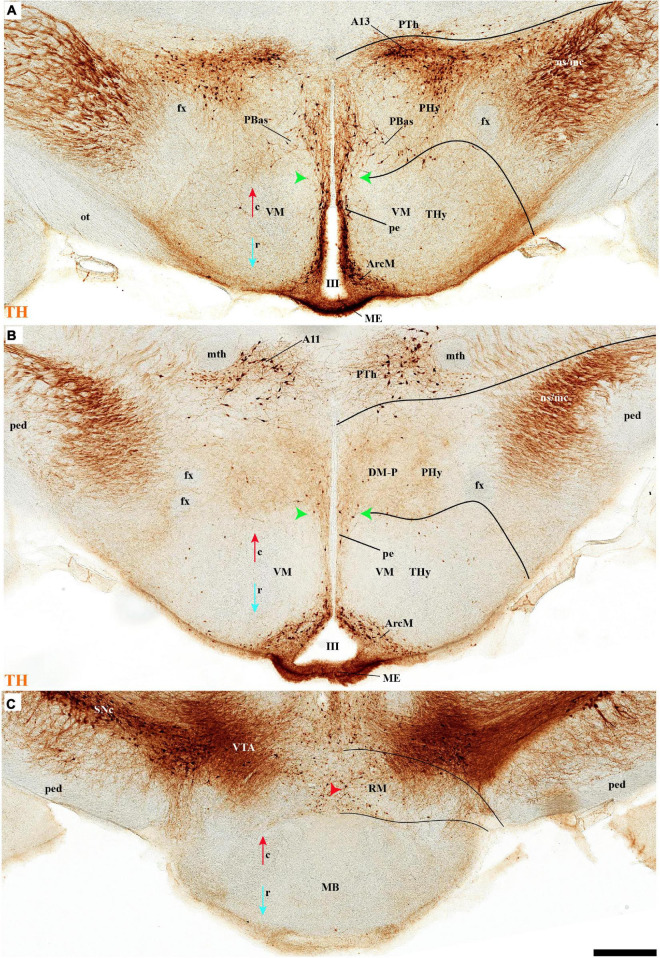
**(A–C)** TH immunohistochemistry highlighting hypothalamic dopaminergic neurons and fibers in selected consecutive horizontal sections of an adolescent rat brain (same as in Figure 5). These sections pass through the basal plate of the hypothalamic region. The peduncular (PHy) and terminal (THy) prosomeric limits (green arrowhead and black lines) are identified. TH-positive neurons are observed in the periventricular stratum (pe), but also in the A13 group and some specific nuclei (PBas, Arc). In addition, some TH-positive neurons are observed in the retromamillary region (RM). For abbreviations see the caption of Figure 1 and Abbreviations List. See section planes in Figure 2A. Orientation arrows: red arrow = caudal; blue arrow = rostral. Scale bar = 500 μm.

The group of TH-positive cells at the pe and deep strata of the PBas nucleus within the RTuD area displays fewer cells than seen at ABas; moreover, this population diminishes shortly before reaching a massive group of dopaminergic cells, known as A13; this is found at the caudal end of PBas, dorsally to the peduncular dorsomedial nucleus (A13; DM-P; Figures 6A, 8A,D, 13A,B, 14A,C and Supplementary Figures 6B, 9A,B,E). The A13 Group was classically ascribed to the prethalamus/zona incerta complex (Chan-Palay et al., 1984; Ruggiero et al., 1984; Van den Pol et al., 1984), but Puelles et al. (2012a) argued its location within the hypothalamus (see Discussion). This very distinct cell group of TH-positive cells consists of a dense core, lying in transversal sections like an isolated island within the intermediate stratum of the caudal PBas (RTuD). It is surrounded by a sparser shell of TH cells all around, but mainly dorsal to the core, possibly lying partly within the overlying alar SPa (Figure 8). Caudally, A13 contacts with the less massive diencephalic dopaminergic A11 cell group. The A13 core nucleus displays no periventricular TH cells; the local pe and deep strata are constituted by TH-negative cells, part of which express selectively *Mch* (Figure 14C). This suggests that the A13 coincides topographically with the source of the MCH cell population within caudal RTuD; some of these peptidergic *Mch* cells also mix with the A13 group and spread radially further (outwards) into the overlying lateral hypothalamus (Figure 14). There is no ependymal layer at the level of group A13 because the ventricle is fused locally (in addition to the more important prethalamic and thalamic fusion). Moreover, a decussation of TH-positive A13 axons can be seen at this location (Figures 6A, 8C,D and Supplementary Figure 9E) (Puelles et al., 2012a).

#### TuI/RTuI

The next basal hypothalamic region we will examine is the tuberal and retrotuberal intermediate domains (TuI; RTuI), which contain the terminal and peduncular parts of the dorsomedial nucleus (DM-T; DM-P) plus the migrated ventromedial nucleus (VM; Puelles et al., 2012a), and ends rostrally in the acroterminal tuberal intermediate domain, which displays the arcuate nucleus, the median eminence, and the hypophysial stalk and gland.

**FIGURE 7:**
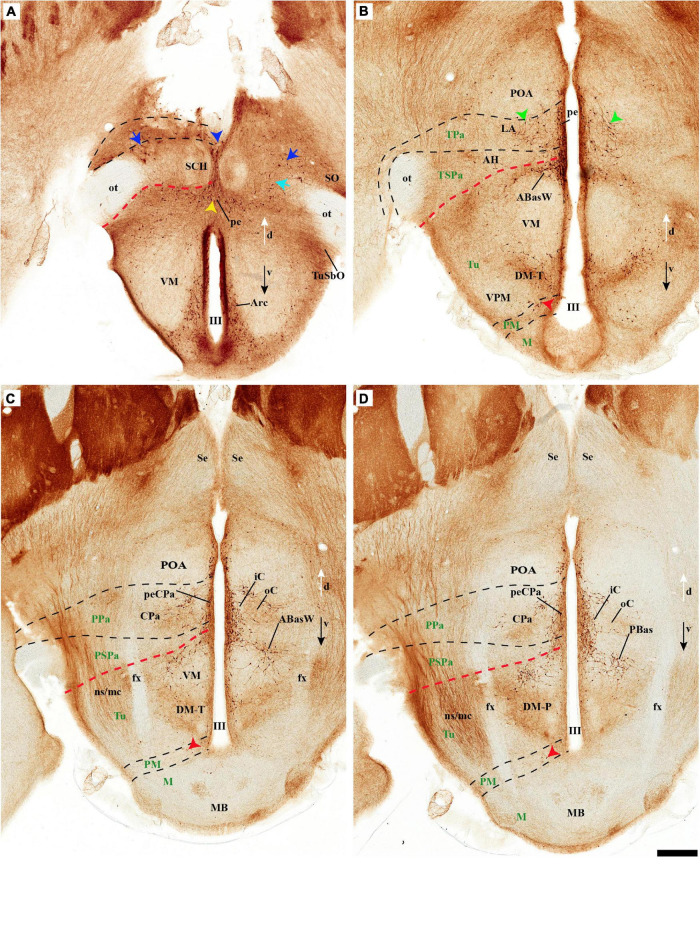
**(A–D)** TH immunohistochemistry highlighting the dorsoventral distribution of hypothalamic dopaminergic neurons in selected transversal sections of an adolescent rat (brain cut parallel to the IHB). This rostrocaudal series (over Figure 8) identifies, from rostral to caudal, the mayor alar and basal plate hypothalamic domains in the terminal (THy) and peduncular (PHy) hypothalamic portions. TH-positive neurons are observed in the periventricular stratum (pe). Some TH-positive neurons are observed in the pe layer of the acroterminal paraventricular (blue arrowhead) and dorsal tuberal subdomains (yellow arrowhead), in the lateral anterior nucleus (LA) of the terminal paraventricular region (TPa) (green arrowhead, **B**), but also in the superficial layers of the TPa and terminal subparaventricular (TSPa) regions (blue arrow and pale blue arrow). In addition, some TH-positive neurons are observed in the medial stratum of the premamillary region (PM) (red arrowhead, **B–D**). The diverse lines follow the code used in Figure 2. For abbreviations see the caption of Figure 1 and Abbreviations List. See section planes in Figure 2A. Orienting arrows: white arrow = dorsal; black arrow = ventral. Scale bar = 500 μm.

The number of TH cells decreases significantly at the TuD/TuI transition at the level of the VM nucleus, which lies within the deep (medial hypothalamic) stratum of TuI (see below; Figures 6A,B, 7A,C, 11A and Supplementary Figure 7A). The local pe layer deep to the VM displays indeed relatively few TH cells (Figures 2A,B, 6B, 7B,C and Supplementary Figures 7A, 8I–L,N–P). In contrast, the neighboring acroterminal pe layer related to the arcuate nucleus shows a dense population of TH neurons, which also exists abundantly through irregularly within the Arc nucleus (Figures 6A,B, 7A, 13A,B, 14A and Supplementary Figures 6, 7, 8A–H,J,M). The Arc complex seems to have a characteristic radial glial texture with deep, intermediate, and superficial mantle strata where different cell populations adopt particular positions. For instance, *Pomc* cells distribute mainly to the deep and intermediate strata, and most abundantly to the intermediate one, where we find intermixed TH cells (ArcM; Supplementary Figure 6A,D, 7A,B). Other *Pomc* cells disperse into the neighboring Arc wing domain (ArcW), thought to lie outside the acroterminal domain within the intermediate stratum of TuI and showing much less TH-positive neuropile (ArcW; Supplementary Figures 6B,G, 7A). *Sst*, *Npy*, and *Agrp* cells appear largely massed at periventricular and deep levels (Supplementary Figures 7C–E), whereas *Cart* cells are distributed through all radial strata of the Arc nucleus (Supplementary Figure 7F). The superficial stratum of the Arc has scarce TH-positive neurons mixed with scarce *Pomc* or *Sst* expressing neurons (Supplementary Figures 7A,B, 8A,C,D). The median eminence and infundibulum are devoid of TH cells while appearing densely filled by TH-positive terminals (Figures 6A,B, 13A, 14A and Supplementary Figure 7A).

Caudal to the median eminence and the Arc nucleus there appears deeply within TuI the terminal part of the DM nucleus (DM-T), ventrally to the VM, and dorsally to the thin ventral tuberal domain (TuV; Figure 1B). This DM-T complex (divided into core and shell parts) is continuous caudally with the peduncular part of DM (DM-P) within RTuI, ventrally to PBas, and dorsal to the ventral retrotuberal domain (RTuV; Figure 1B). The lateral and superficial stratum of the DM-T is invaded by the ventral premamillary nucleus (VPM), which migrates out of the retromamillary area (Puelles et al., 2012a; López-González et al., 2021; Supplementary Figures 6B,C,F,G). The pe stratum found deep to the DM-T shows abundant TH-positive cells, which decrease gradientally in number caudalwards (minimum at the DM-P). TH-positive cells are mainly found dispersed in the DM-T shell domain, which is also pervaded by TH-positive neuropil (Figures 2A,B, 3A, 7B,C, 11A, 13A,B, 14A and Supplementary Figure 6A); some of these TH cells even protrude superficially between the TH-negative VM and VPM nuclei (Figure 13B). The peduncular DM nucleus also shows similar core and shell portions, the latter likewise displaying some dispersed TH cells and general TH-positive neuropil in its core (Figures 13A,B, 14A,B and Supplementary Figures 9A,B). There is a triangular space between the DM-P and the VM nuclei, with its base placed in the ABas-PBas transition, where large magnocellular Trh neurons are observed, jointly with additional TH-positive neurons (Figures 2A,B, 6A, 7D, 13B–B′ and Supplementary Figures 9A,B,D,E).

#### TuV/RTuV

The ventralmost longitudinal domain of the tuberal-retrotuberal basal plate (TuV/RTuV) is a thin band intercalated between TuI/RTuI and PM/PRM where some cells express *Mch* and *Hdc* mRNA (Puelles et al., 2012a); we did not observe TH-positive cells at this locus (Figure 14B).

#### Perimamillary/Periretromamillary

**FIGURE 8:**
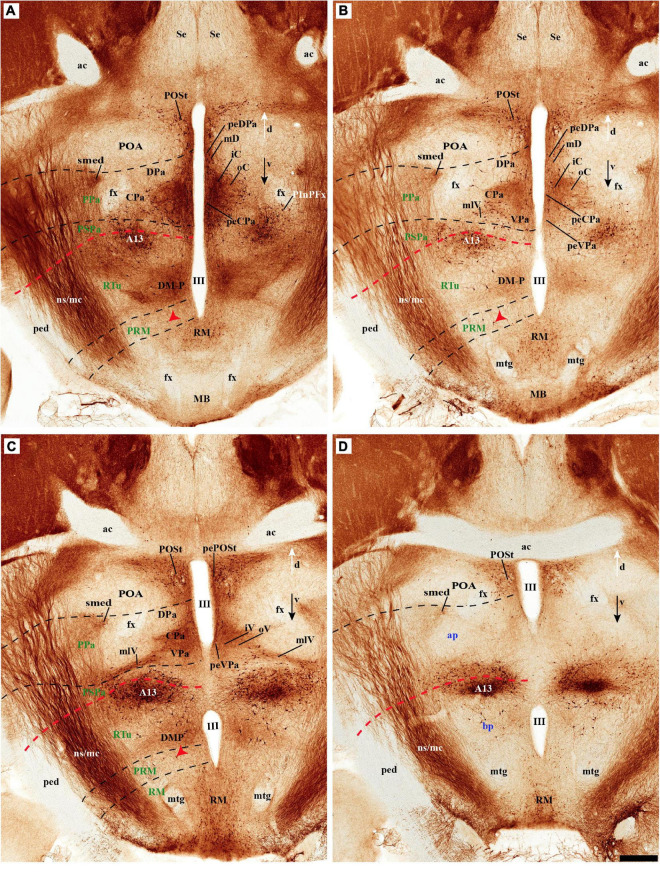
**(A–D)** TH immunohistochemistry highlighting more caudal dorsoventral parts of the hypothalamic prosomeres than in Figure 7, in selected transversal sections of an adolescent rat brain. TH-positive neurons are observed mainly in the pe stratum of the retromamillary region (RM), the pe and medial strata of the Pa nucleus, and in the A13 group. In addition, some TH-positive neurons are observed in the medial stratum of the periretromamillary region (PM) (red arrowhead, **A–C**). TH-positive neurons are observed in the POSt cell group of the preoptic region **(A,B)**. The diverse lines follow the code used in Figure 2. For abbreviations see the caption of Figure 1 and Abbreviations List. See section planes in Figure 2A. Orienting arrows: white arrow = dorsal; black arrow = ventral. Scale bar = 500 μm.

The PM domain produces the dorsal premamillary nucleus, whereas the PRM corresponds to the classic intrahypothalamic part of the ‘posterior hypothalamus’; this region typically expresses *Otp* and *Sim1* genes (Shimogori et al., 2010; Puelles et al., 2012a). There are some scattered TH-positive cells in the medial stratum of this region (Figures 2A,B, 3A, 7B–D, 8A–C, 13A,B, 14A,B and Supplementary Figures 6A,B,G, 9A,B). On the other hand, few such cells were observed in the medial and superficial lateral strata of the PRM region (Figures 2A, 7C,D, 14A and Supplementary Figures 9A,B).

#### Mamillary/Retromamillary

The M domain forms the whole mamillary body and is practically devoid of TH-positive cells, though scarce TH-positive cells can be found scattered in the mamillary portion of the acroterminal domain, sometimes defined as the median part of the mamillary nucleus (Figures 2A,B, 6C, 7C,D, 8A,B, 13A,B, 14A,B and Supplementary Figures 6A,B, 9A,B). However, the number of TH-positive cells increases substantially in the periventricular and medial retromamillary nucleus (RMM), as well as in the lateral retromamillary nucleus (RML) of the RM domain (Figures 2A,B, 6C, 7C,D, 8A,B, 13A,B, 14A and Supplementary Figures 6A,B, 9A,B). In addition, a caudolateral part of the RML area shows a distinct small group of large TH-positive neurons. These lie just in front of the diencephalic compact substantia nigra, and may represent a minor PHy contribution to the latter plurineuromeric (mesodiencephalic) dopaminergic formation (Figures 4A,B).

## Discussion

### The Alternative Columnar and Prosomeric Models of the Hypothalamus

Most studies defining the hypothalamic region and its nuclei during the last century followed the *columnar model* (e.g., Herrick, 1910; Kuhlenbeck, 1927, 1973; Bleier, 1979; Swanson, 1987; Armstrong, 2004). Likewise, a large part of the research projects aimed at hypothalamic neurochemical mapping, connectivity, and physiology in rats were guided by the columnar paradigm (Swanson, 1987, 2003; Canteras et al., 1992, 2011; Elias et al., 1998, 1999; Torrealba et al., 2003; Armstrong, 2004, 2015; Choi et al., 2005; Yoshida et al., 2005; Markwell et al., 2010). In its origins, the columnar model proposed by Herrick (1910) aimed to extrapolate into the forebrain the theory of hindbrain *columnar* sensorimotor and viscerosomatic functional compartmentation developed after the discovery of longitudinal neuronal columns associated with specific cranial nerve fiber components (Gaskell, 1889; Johnston, 1902; Herrick, 1903). To this end, Herrick redefined *de facto* and without discussion the length axis of the forebrain. He departed from the earlier axial reference defined by His (the *sulcus limitans* separating neurogenetically heterochronic alar and basal plates; His, 1893, 1904). Herrick reinterpreted three other diencephalic ventricular sulci that intersected obliquely His’ longitudinal sulcus limitans as ‘longitudinal’ landmarks held to define four diencephalic columns analogous to the hindbrain ones (see Puelles and Rubenstein, 2015; their Figure 2). The consequently redefined hypothalamus (since that of His was restricted to the basal region ventral to sulcus limitans; see the historic account of the hypothalamus concept in Puelles et al., 2012a) was the ventralmost diencephalic column within the new “longitudinal four”: epithalamus, dorsal thalamus, ventral thalamus and hypothalamus (Herrick, 1910). Each of these *diencephalic longitudinal columns* supposedly ending in the telencephalon (soon shown to be untrue) was assumed to be structurally and functionally *homogeneous*, akin in this aspect to the hindbrain columns (Herrick, 1948; Kuhlenbeck, 1973).

However, 100 years later, the hypothalamic region turns out to be a structurally and functionally non-homogeneous region. The last three decades of progress in understanding the molecular map and fate of hypothalamic parts (Puelles and Rubenstein, 1993, 2003, 2015; Puelles et al., 2012a; Ferran et al., 2015a; Díaz and Puelles, 2020) increasingly suggest inconsistency of the molecular data with the columnar model, and agreement, instead, with the old axial notion of His (1893, 1904), as well as with earlier forgotten neuromeric studies (Puelles et al., 1987; Puelles, 2021). For instance, the *Nkx2.2* gene is expressed in all vertebrates studied (both in embryos and adults) in a thin band along the alar-basal boundary all the way from the isthmus to the retrochiasmatic hypothalamic region, roughly as predicted by the sulcus limitans of His (see Puelles and Rubenstein, 2015; their Figure 7); this longitudinal band *crosses* all the diencephalic ‘longitudinal’ sulci of Herrick, and does not enter the telencephalon, contrary to what was predicted for the alar-basal boundary by the columnar model (discussed in Puelles et al., 2012a; Puelles and Rubenstein, 2015). Many detailed molecular hypothalamic subdivisions appeared which are not explained by the columnar model but can be understood easily as either neuromeric AP or dorsoventral (DV) compartmentation (Puelles and Rubenstein, 2003; Shimogori et al., 2010; Morales-Delgado et al., 2011, 2014; Puelles et al., 2012a; Ferran et al., 2015a; Puelles, 2017). This progress laid the foundation for the proposal of the alternative *prosomeric brain model* (see an account of its origin in Puelles, 2021). This model is based on the definition of transverse neuromeres as serial AP developmental units that display a shared organization into longitudinal DV floor, basal, alar, and roof zones or plates (Figures 1A,B).

The currently updated *prosomeric model* is consistent with masses of detailed molecular data obtained from different vertebrates (Puelles and Rubenstein, 1993, 2003, 2015; Martínez et al., 2012; Puelles et al., 2012a,c; Puelles and Ferran, 2012; Nieuwenhuys and Puelles, 2016; Moreno et al., 2017; Nieuwenhuys, 2017; Puelles, 2018, 2019, 2021). Studies of genetic patterning, regionalization, and gene expression profiles have led to a precise, causally underpinned definition of the prosomeric forebrain axis (thus defining causally, rather than arbitrarily or by convention, what is longitudinal in the brain) and several anatomical fiber tract landmarks that co-define the hypothalamic transversal boundaries (e.g., the supraopto/paraventriculo-hypophysial tract, the fornix tract and the medial and lateral forebrain bundles of the cerebral peduncle; Keyser, 1972; Puelles et al., 1987, 2012a; Bardet, 2007; Bardet et al., 2008; Puelles and Rubenstein, 2015). Gene expression patterns in mice likewise identified a variety of hypothalamic alar and basal plate subdivisions, which are not accounted for by the columnar model, not only in the mouse (Bardet et al., 2008; Shimogori et al., 2010; Morales-Delgado et al., 2011, 2014; Puelles et al., 2012a; Díaz et al., 2015; Ferran et al., 2015a; Puelles and Rubenstein, 2015), but also in chicken (Bardet, 2007; Bardet et al., 2008), frog (Bardet et al., 2008; Domínguez et al., 2013, 2014; González et al., 2020), reptiles (Moreno et al., 2012, 2017; Domínguez et al., 2015), catshark (Santos-Durán et al., 2015, 2016, 2018) and zebrafish (Lauter et al., 2011; Schredelseker and Driever, 2020). The adult structural and hodological organization of the hypothalamus of vertebrates thus appears to be the result of causal patterning mechanisms active during the development of this region with regard to the axial dimension first defined by His.

Most studies in the hypothalamic region in rats nevertheless followed the insufficient columnar model. In this model, the hypothalamic region is the ventralmost diencephalic longitudinal domain, separated from the ventral thalamus (prethalamus in the prosomeric model) by the ventral diencephalic sulcus (Herrick, 1910; see schemata of variant columnar models in Puelles et al., 2012a). A ventral (basal) longitudinal position of the hypothalamus is thus assumed, jointly with anteroposterior subdivisions into preoptic, anterior, tuberal, and mamillary/posterior hypothalamus regions (Figure 15A). In addition, some studies identified dorsal, intermediate, and ventral hypothalamic partitions, with the dorsal part supposedly bordering the ventral thalamus (Swanson et al., 1981; Chan-Palay et al., 1984; Ruggiero et al., 1984; Van den Pol et al., 1984; note Puelles et al., 2012a concluded that the classic dorsal hypothalamus structures actually fell *within* the prethalamus or ventral thalamus, and were thus not hypothalamic unless they migrated therefrom; see below some evidence that this may occur at the cellular level). The columnar intermediate and ventral ‘longitudinal’ hypothalamic parts roughly correspond to the prosomeric *transverse* Phy and THy parts, respectively.

According to the prosomeric model, the hypothalamus is instead, topologically, a *rostral* region of the brain bent morphogenetically into a ventral topography, due to the constant axial bend at the cephalic flexure. The hypothalamus is located primarily in the secondary prosencephalon, *rostral* to the diencephalon proper and *ventral* to the telencephalon, which is held to be a dorsal evagination from the alar hypothalamus (Figures 1A,A′; Puelles et al., 2012a; Puelles and Rubenstein, 2015; Ferran et al., 2015a; Nieuwenhuys and Puelles, 2016). The preoptic area is modernly not regarded as part of the hypothalamus but represents instead a non-evaginated subpallial telencephalic region (a.k.a. as *telencephalon impar*), as was thought originally in the time of His (POA in Figure 1A; Gurdjian, 1927; Crosby and Woodburne, 1940; Bardet et al., 2010; Puelles et al., 2012a,^2013^, ^2016^). The sub-telencephalic transversal hypothalamic region develops a large number of individual nuclei or dispersed cell populations derived from either the alar or basal plate domains of the hypothalamic prosomeres 1/2 (hp1, hp2). These define, respectively, a caudal *peduncular hypothalamus* that extends dorsally into the evaginated telencephalon (Phy; hp1; soft pink; Figures 1A,B) and a rostral *terminal hypothalamus* continuing into the non-evaginated preoptic area (THy; hp2; stronger pink; Figures 1A,B; Puelles et al., 2012a; Puelles and Rubenstein, 2015). The prefix tags ‘P’ and ‘T’ are used generally to refer to the fundamental Phy/THy division.

The prosomeric concept of the floor, basal, alar, and roof plates differs somewhat from that of His (1893, 1904). The bilateral ridge that delimits the open neural plate from head skin tissue is defined in accord with fate-mapping experiments as representing the prospective fused and thus median *dorsal roof plate* of the neural tube and brain. The roof plate of the closed neural tube ends rostrally at the septo-preoptic median bed of the anterior commissure (Figures 1A,A′; fate maps of Puelles et al., 1987; Cobos et al., 2001; Inoue et al., 2000; note His’s roofplate was shorter). The prosomeric *ventral floorplate* stops short of that neural plate ridge, due to its strict co-extensiveness with the underlying notochord, which is its inducer (the floor starts to form by nodal gastrulation of chordal nature caudal to the precocious prechordal plate; see Ferran et al., 2022), whereas the floorplate of His was merely assumed to end at the rostral ridge, similarly, as the basal plate, wrongly holding at the same time that the rostral ridge represents the prospective optic chiasma. The modernly defined anterior end of the floor plate accordingly stops at the mamillary body; see also Puelles et al. (2012a), Puelles and Rubenstein (2015), Puelles (2018).

The different neural plate topology of the prospective roof and floor plates implies that the alar and basal plates (separated by the alar-basal boundary) must reach in parallel a topologically dorsoventral lineal end of the rostral neural tube that is primarily closed and connects along the rostral midline of the neural plate the rostral ends of the floor and roof longitudinal zones. This unique rostromedian territory was defined recently as the ‘acroterminal’ forebrain domain (Aterm; Figure 1B; Puelles et al., 2012a; Puelles and Rubenstein, 2015; Puelles, 2018). The right and left basal and alar plates are thus continuous, respectively, from side to side across the acroterminal region of the terminal prosomere (ATerm; hp2; Figure 1B). The rostromedian derivatives found in the adult brain between the roof anterior commissure and the floor mamillary region include the thin terminal lamina of the preoptic area, the optic chiasma (plus suprachiasmatic nuclei and evaginated eyes), the retrochiasmatic or anterobasal tuberal area, the median eminence and infundibulum, the neurohypophysis, the arcuate nucleus, and the ventral tuberomamillary area, plus an acroterminal part of the mamillary body. All belong in the prosomeric model equally to the *most rostral part of the brain*, representing a median (virtually symmetric) acroterminal DV sequence (Puelles et al., 2012a; Puelles and Rubenstein, 2015; Puelles, 2018).

The molecular and structural patterns observed during the early stages of hypothalamic development indicate that after neural tube closure a fundamental AP regionalization of the hypothalamus takes place, resulting in at close range in the specification of two hypothalamic neuromeric units (hp1; hp2; Figure 1A′), separated by the *intrahypothalamic boundary* (IHB; Figure 1B). The caudal limit of the hypothalamus corresponds to the *hypothalamo-prethalamic boundary* (the thick black line separating pink from yellow territories; Figure 1B); the latter courses just caudal to the cerebral peduncle and in front of the prethalamic reticular nucleus and zona incerta derivatives (Puelles et al., 2012a; Ferran et al., 2015a; Puelles and Rubenstein, 2015).

Hypothalamic morphogenesis, neuronal differentiation, and axonal navigation patterns are held to be influenced causally by morphogens diffusing gradientally from the roof (DV), acroterminal (AP), and floor (DV) midlines; this is reflected in the observed courses of most axonal tracts (Figures 1C,D). Antagonistic signals diffusing from the roof plate (dorsalizing effects of WNTs/BMPs) and the floor plate (ventralizing effects of SHH) are involved early on in patterning the DV regionalization of the Phy, THy, and Aterm hypothalamic parts, creating their alar/basal regions and the related alar-basal boundary, as occurs elsewhere in the brain. In addition to the underlying alar hypothalamic PA and SPa domains (Tpa/Ppa; TSPa/PSPa), these opposed gradients also pattern the telencephalon as an exclusively alar outgrowth. On the other hand, molecularly distinct tuberal/retrotuberal (Tu/Rtu), perimamillary/periretromamillary (PM/PRM), and mamillary/retromamillary (M/RM) domains are patterned as DV parts of the hypothalamic basal plate, stacked dorsoventrally between the alar-basal boundary and the mamillary/retromamillary hypothalamic floor (Figure 1B; Puelles et al., 2012a; Puelles and Rubenstein, 2015; Nieuwenhuys and Puelles, 2016); each pair of denominations refers, respectively, to longitudinally corresponding domains of THy and Phy.

Note that the prosomeric alar Pa region used to be included classically in the preoptic area (as ‘preoptic magnocellular nuclei’), whereas the SPa alar region roughly corresponds to the columnar ‘anterior hypothalamus’ (though this concept is used modernly in a very vague way; comments in Puelles et al., 2012a). The Tu/Rtu basal complex roughly corresponds to the columnar ‘tuberal hypothalamus,’ and the PM/PRM plus M/RM complexes jointly form the columnar ‘posterior hypothalamus/mamillary’ region (compare Figures 1B,C). Obviously, the ontology of what exists in the hypothalamus is largely identical in both models, but individual elements are referred to descriptively with regard to different (actually orthogonal) length axes (e.g., confusingly causing the columnar ‘ventral premamillary nucleus’ to lie *dorsally* to the ‘dorsal premamillary nucleus’ in the prosomeric model, and both ‘premamillary entities’ are found *dorsal* to the mamillary body, rather than rostrally to it).

The prosomeric area map is on the whole more discriminative than the columnar one, since it adds AP and DV subdivisions, and refers to two neuromeres (hp1, hp2; or Phy, THy) plus the acroterminal domain (Aterm). As a result, there is a checkerboard pattern of subregions wherein specific cell populations can be pinpointed. The clearcut AP/DV pattern and attached morphogenetic signaling possibilities also introduce the possibility of causal explanations as a result of theoretically invoked DV and AP patterning mechanisms originated, respectively, from the roof and floor versus the ATerm (Puelles, 2017; Díaz and Puelles, 2020).

### The Prosomeric Pattern of Hypothalamic Tyrosine Hydroxylase Groups

Theoretically, any spatial arrangement of neuronal populations in the brain may be explained causally by analysis of the data with regard to a morphologic *model* that (a) embodies a consistent set of assumptions about axial, dorsoventral, and anteroposterior dimensions, (b) maps consequently relevant regional subdivisions, and (c) postulates the relative positions of potential secondary organizers of the local pattern (i.e., relevant sources of patterning morphogens). We have attempted to perform this task for hypothalamic dopaminergic cell populations, examining their 3D positions relative to the dimensional assumptions, the hypothalamic subdivisions, and the potential secondary organizers postulated in the updated *prosomeric forebrain model* (Puelles et al., 2012a,c, 2013; Ferran et al., 2015a; Puelles and Rubenstein, 2015; Puelles, 2017, 2018, 2021; Díaz and Puelles, 2020; Amat et al., 2022). We have attempted to frame the histological data within the prosomeric model, and merely discuss briefly here some interpretive possibilities, believing that this approach may be of interest in the long run for those looking for causal explanations for related neuroanatomic structure. Obviously, further studies and experimental tests are needed to conclude scientifically about specific testable causal hypotheses for each of the dopaminergic cell groups.

In retrospect, it may be reflected that the alphanumeric classification of catecholaminergic cell groups advanced by Dahlström and Fuxe (1964) and further elaborated by various followers (see Introduction and Figure 15A) may have been the result of difficulties encountered in the classification of these cell groups within the then prevailing *columnar forebrain model* of Herrick (1910), Herrick (1933), Herrick (1948) and Kuhlenbeck (1927, 1973). The Nordic authors possibly discovered oddities or inconsistencies in their likely initial effort to ascribe their histochemically mapped cell groups to the postulated system of *brain vesicles and columns* of the columnar model then absolutely prevalent. For instance, several of the studied cell groups–including the A9, or A11 groups, for example–appear *elongated* along a dimension that is incoherent with the direction of columnar longitudinal sulci and columns. Remarkably, there exist no columnar descriptions or flat maps of the multiple brain CA cell groups (i.e., descriptions detailing column by column their content of catecholaminergic cells). Perhaps noticing the absence of a satisfactory model, Dahlström and Fuxe (1964) and followers turned to the pragmatic alphanumeric solution. During the last 60 years, this classification has been widely used in the field, unwittingly representing an aberrant result of anatomic analysis, insofar as it implicitly contradicts *without saying so* the then prevailing columnar brain model (incapable to account for the pattern). Unfortunately, this also tended to preempt efforts toward causal explanations, since the chosen arbitrary denominational solution is devoid of a contextual causal framework. In a hypercomplex system as is the brain, empiric data *unrelated* to a structural model or its developmental assumptions do not have explanations or morphologic meaning. Of course, a model-based interpretation is necessarily based on assumptions. A specific model provides an informed and useful interpretation coherent with various aspects of the data, but not the truth; the potential long-term validity of such interpretations depends on the historic development of the science (kinds of available data needing explanation) and how long the models’ interpretive usefulness prevails subjectively.

In this sense, our present effort thus attends to a probably still unrecognized old neuroanatomic problem: can we encompass the observed distribution of brain dopaminergic neurons (or just the hypothalamic ones) within a theoretic framework of spatial references and developmental phenomena – a model- that provides a credible explanatory causal background? The option to try the alternative and relatively novel *prosomeric model* toward achieving this generic aim was actually explored earlier with previous, less elaborate versions of this model. Indeed, L Puelles and collaborators attempted several times in the past to insert the conventional alphanumeric groups of catecholaminergic neurons within the prosomeric model in avian, mouse, and human embryonic brains. They had mixed success due to some problematic assumptions held at the time that needed to be corrected subsequently (Puelles and Medina, 1994; Puelles and Verney, 1998; Marín et al., 2005; Puelles et al., 2012a,c). Moreover, Puelles covered recently the scenario of known or possible secondary organizer sites existing in the forebrain (Puelles, 2017). Separately, Marín (Marín et al., 1998; their Figure 3) mapped schematically mesodiencephalic dopaminergic cell groups within the prosomeric model in tetrapods.

The main impact of this earlier prosomeric interpretive work so far was to convince several specialists on catecholaminergic neurons of the existence of a *mesodiencephalic plurineuromeric constitution* of the classic VTA and the SNC, a pattern already captured by the early prosomeric model (Puelles and Medina, 1994; their Figure 16.6B). In the columnar model, the same DA system is devoid of serial parts, and all the diencephalic components are ascribed rather arbitrarily to the midbrain. The alternative prosomeric interpretation visualizes a *longitudinal* tegmental DV differentiation pattern that is repeated with minor changes across several neuromeric units (midbrain m1–m2, and diencephalic p1–p3 in the updated model). The SNC/VTA dopaminergic phenotype seems to respond to potential close-range ventralizing floorplate/notochordal morphogenetic signals that are repeated along with these five modules. This mesodiencephalic neuromeric model background subsequently led to the identification of a floorplate-related gene cascade that operates serially to produce different features of these particular dopaminergic neurons (e.g., Smits et al., 2006). Not all forebrain dopaminergic neurons admit the same explanation, though, so we must conclude that other ranges of action, or other causal molecular DV and/or AP circumstances also deducible from the prosomeric model may lead to an *analogous* dopaminergic cell type elsewhere within the neural checkerboard pattern. An example of differences in dopaminergic identity is seen in meso-diencephalic dopaminergic cells, of midbrain and diencephalon (p1, p2, and p3) origin, which express dopamine active transporter (DAT), while this is absent in hypothalamic dopaminergic neurons (Sánchez-González et al., 2015). In the molecular era, one cannot expect the alar pretectal DA neurons to be patterned, similarly, to the midbrain VTA DA neurons. For this reason, careful topologic classification of hypothalamic TH cells is a prerequisite for the ulterior meaningful search of the probably combinatorial causes of each particular TH cell group that differs significantly in relative position.

The present analysis concentrated on the hypothalamic pattern of TH-positive neurons. This was studied previously with the same model, but in less detail (Puelles et al., 2012a). In their mapping of dopaminergic neurons in the mouse hypothalamus (based only on sagittal sections), Puelles et al. (2012a) reported basically widespread periventricular A14 TH-positive cells in the alar PA and SPa domains (with added subpial supraoptic cells), and four groups of basal plate dopaminergic neurons including the A13 group, the dorsal and ventral tuberal groups, and the arcuate A12 group. We examined the same territory in continuous series cut in three section planes (sagittal, horizontal, and transversal to the prosomeric axis through the hypothalamus). Note that our horizontal sections are parallel to the optic tract, being thus similar to conventional coronal atlas sections. They accordingly reveal a series of AP relationships. Despite our transversal hypothalamus sections being less commonly used, they otherwise illuminate DV relationships (they are essentially parallel to both the acroterminal domain and the cerebral peduncular and nigrostriatal tracts, and orthogonal to the optic tract). We further mapped by ISH the relative positions of several peptidergic cell populations found in the neighborhood of dopaminergic neurons.

An extensive alar periventricular stratum of TH-positive cells (possibly subdivided into Pa and SPa domains) was corroborated in the present analysis. We further distinguished several groups of TH neurons intermixed with peptidergic neurons in the neighboring medial hypothalamic stratum, both at the PA and SPa grisea. The PA complex shows more significant TH populations within PHy, whereas the TH cells of SPa mainly appear within the anterior hypothalamic nucleus within THy (Figure 15B). These differences may bespeak differential AP patterning (hp2 versus hp1), apart from the DV step existing between PA and SPa. Within the peduncular PA nucleus (subdivided between the model into dorsal, central, and ventral components; Puelles et al., 2012a) we noted inner and outer nuclear strata invaded by some TH cells (particularly at the Cpa), as well as similar partial TH subpopulations within the discontinuous inner, outer, mediolateral and laterolateral parts of the Vpa. Note standard columnar terminology identifies our Vpa as ‘caudal Pa’ (due to the orthogonal axis used). Our Dpa component (similar to columnar Apa) showed very sparse TH cells. The presence of TH neurons within the Pa did not seem to correlate specifically with any of the peptidergic cell types we mapped therein (see Results), though these did show themselves some already described specificity for given parts of the Dpa, Cpa, Vpa subnuclei. The conventional columnar ‘dorsal Pa’ subgroups (which also show some mixed TH cells), often ascribed to the ‘dorsal hypothalamus,’ were interpreted within our model as *lying strictly within the deep strata of the prethalamus*. We accordingly renamed them *caudal Pa* by reference to the prosomeric axis. It may be conjectured that these cPa neurons have migrated out of early PA hypothalamic origins into the neighboring compressed prethalamic domain (see the intercalated position of a thin wedge of GABAergic prethalamic territory between glutamatergic thalamic and PA territories in Puelles et al., 2012c; their Figures 9.4, 9.5).

**FIGURE 9:**
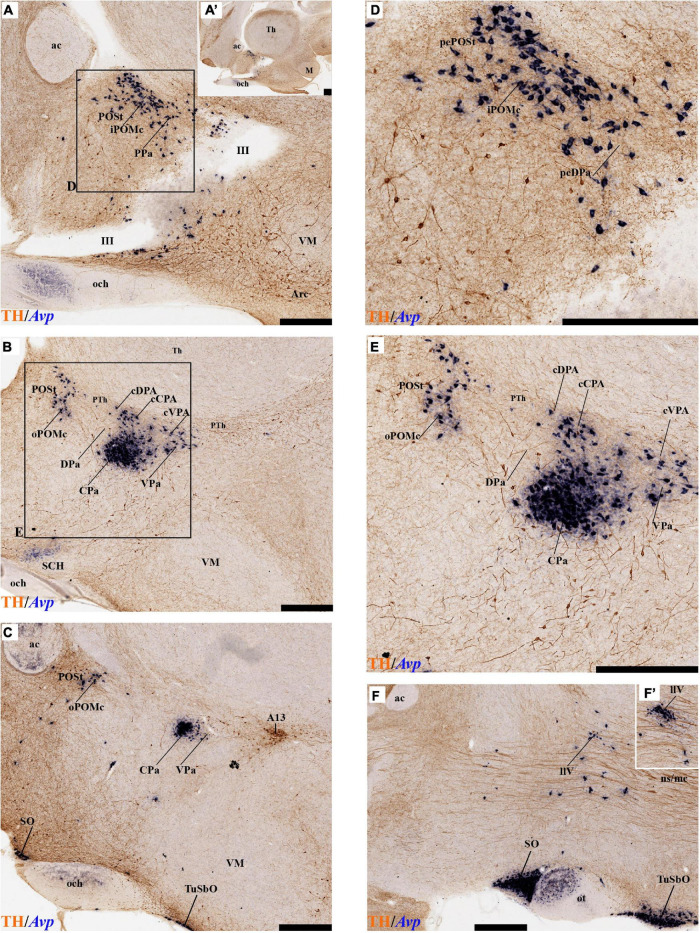
**(A–F′)** Combined TH immunohistochemistry and *Avp* ISH in selected consecutive sagittal sections of an adolescent rat brain. (**A,A′,D =** higher magnification of **A**) Periventricular section showing *Avp* expression in the preoptostrial nucleus (POSt) and neighboring DPa. (**B,C,E** = higher magnification of **B**) Selected sagittal sections through the medial hypothalamic stratum identifying *Avp* expression in POSt, CPa, and VPa. **(F,F′)** Selected sagittal section level with the lateral VPa stratum, identifying scattered or aggregated *Avp* cells of the llV component within the ns/mc tract. Superficially, *Avp* cells also characterize the SO and TuSbO nuclei. For abbreviations see the list. See section planes in Supplementary Material 4. Scale bar = 500 μm.

The subpial position of the *Avp*-rich supraoptic nucleus and its accompanying TH cells along with the terminal Pa and just above the optic tract is a phenomenon unique in the hypothalamus and may require a singular explanation. It is to be noted that various neurogenetic data indicate that the hypothalamic mantle develops outside-in order. Curiously, SO cells do not exist at Phy levels occupied by the cerebral peduncle. Interestingly, there appears at Phy section levels corresponding to the Cpa and Vpa a small rounded mass of relatively lateralized *Avp* neurons adjacent medially to the ns/mc tract, which we named, respectively, *laterolateral central* and *laterolateral ventral* cell groups (llC; llV; Figures 9F,F′, 11C,E,E′). They probably correspond to what Peterson (1966) called the ‘accessory PA cell group.’ We now think it is possible that they may represent somewhat younger ectopic SO cells born after the peduncle (and the mfb) started to form superficially at the Phy surface, thus being secondarily separated from the subpial SO elements. Remarkably, Puelles et al. (2012a) alerted to transient data suggesting that some SO neurons appear to migrate ventralwards just deep to the optic tract, collecting thereafter subpially at the tuberal surface, ventrally to the optic tract, in the classic ‘tuberal supraoptic nucleus,’ which is obviously a partial topographic misnomer. It was suggested to identify them as a *tuberal suboptic population*. We did not notice significant numbers of TH cells at this secondary site.

**FIGURE 10:**
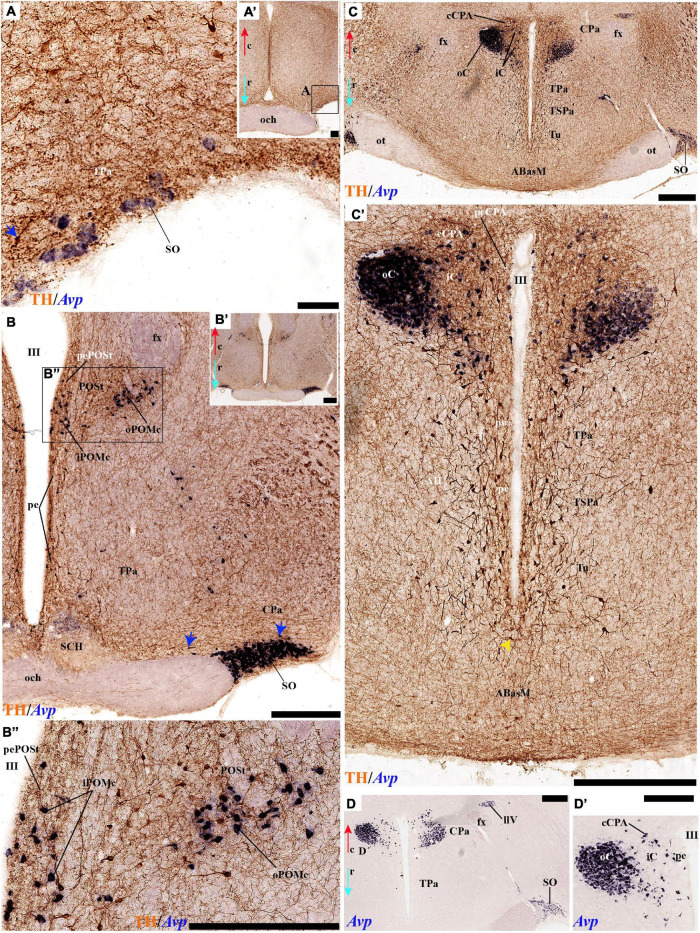
**(A–D′)** Combined TH immunohistochemistry and *Avp* ISH in selected consecutive horizontal sections through alar hypothalamic domains of an adolescent rat brain. Expression of *Avp* mRNA is observed in the SO nucleus **(A,A′)**, POSt nucleus (upper left corner; **B–B″**; see also SO nucleus), and CPa region **(C,C′,D,D′)**. The combined expression of TH and *Avp* helps to recognize different radial portions in the CPa. For abbreviations see the list. Orientation arrows: red arrow = caudal; blue arrow = rostral. Scale bar = 500 μm.

**FIGURE 11:**
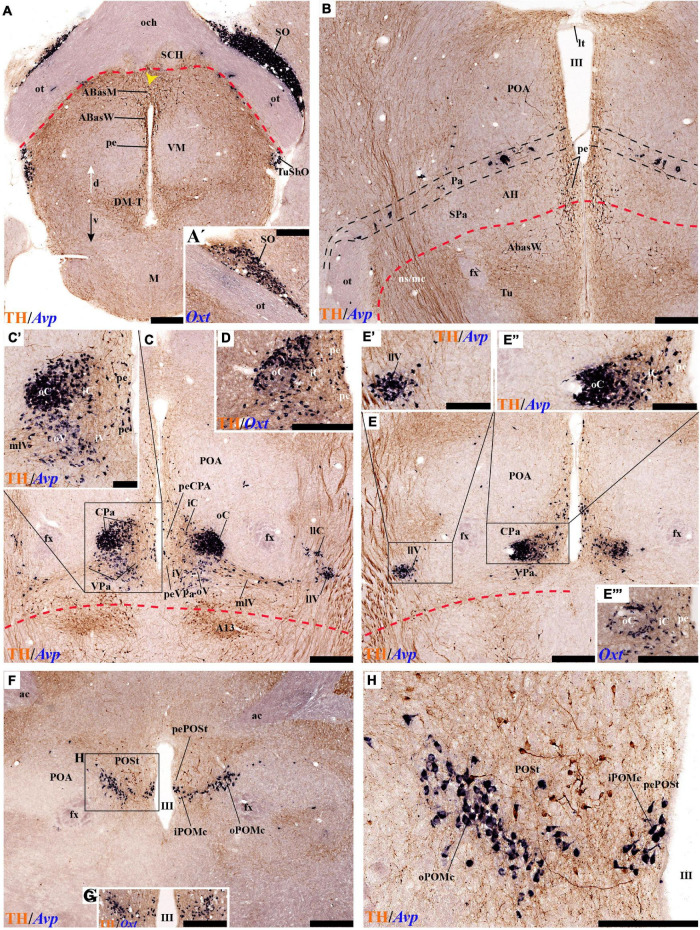
**(A–H)** TH immunohistochemistry combined with *Avp in situ* hybridization in selected transversal hypothalamic sections of an adolescent postnatal rat, illustrating details of alar and basal domains, and with insets comparing correlative *Oxt* signal. A rostral section through the upper tuberal region and the optic chiasma identifies *Avp* in the SO and TuSbO nuclei, as well as TH cells in the acroterminal TuD, the TuD, and the DM-T areas (ABasM; ABasW; DM-T; pe; **A**; the inset **A′** shows *Oxt* signal at the SO). A more caudal section through the tuberal and preoptic regions (Tu; POA; **B**) shows TH cells in diverse alar and basal periventricular domains (POA; PA, SPA, and TuD) and the TH-positive ns/nc tract within the lateral hypothalamus. Note *Avp* cells are present mainly within the alar Pa domain, at various distances from the ventricle. **(C)** a section passing through the POA and the Pa complex (CPa/VPa; iC, iO, iV, oV; peVPa, peCPa), as well as the underlying basal A13 dopaminergic nucleus, illustrates periventricular, medial, and lateral peptidergic subpopulations, leading through mediolateral cells (mlV) into laterolateral llC and llV *Avp* cell groups (**C′** shows a higher magnification of the Pa complex, while **D** shows local *Oxt* signal). **(E)** Adjacent section illustrating a larger clump of *Avp* cells at the llV locus (**E′** inset: higher magnification detail of llV; **E″′**: higher magnification detail of CPa). The most caudal transversal section shows the *Avp* and *Oxt* populations present at the ventral border of the POSt nucleus (**F–H** = higher magnification of box in **F**). Alar-basal boundary in red. For abbreviations see the list. Orienting arrows: white arrow = dorsal; black arrow = ventral. Scale bar = 500 μm.

**FIGURE 12:**
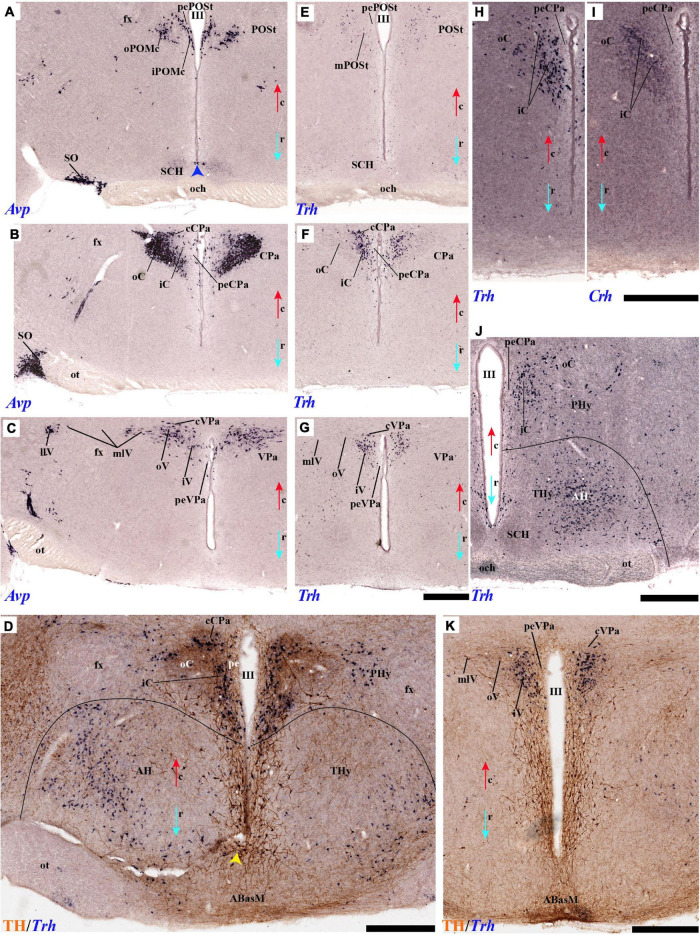
**(A–C,E–G)** Comparison of *Avp* and *Trh* ISH signal at three horizontal (coronal) section levels. **(A,E)** lie rostral to **(B,F)** and **(C,G)**, and all levels interest the alar plate. Each marker shows characteristic (unequal) aggregates of labeled neurons occupying positions close or separated from the ventricle. At **(A,E)** the POSt area shows its subdivisions. **(B,F)** displays the CPa complex. **(C,G)** passes through the VPa complex. **(D)** TH combined with *Trh* ISH in the Pa area; note PHy versus THy differences. **(H,I)** Comparison of *Trh* and *Crh* ISH on adjacent horizontal sections through alar plate domains highlighting CPa populations. **(J)** Similar horizontal section level with the optic chiasma, showing *Trh* ISH identifying CPa and AH derivatives. **(K)** TH immunoreaction combined with *Trh* ISH in a horizontal section across the alar-basal border, showing VPa positive cells and the periventricular TH elements. For abbreviations see the list. Orientation arrows: red arrow = caudal; blue arrow = rostral. Scale bar = 500 μm.

In our study of the alar plate, we also noted TH cells in the preoptic area. This is interpreted in the prosomeric model as a telencephalic subpallial field included dorsally to THy within the hp2 prosomere. According to the historical summary offered in Puelles et al. (2012a), classic neuroanatomy first held this territory to be telencephalic and often included within it the subjacent PA area; later, after the nineteen forties, it became customary to include the preoptic area in the rostral part of the columnar hypothalamus. After the advent of molecular studies at the end of the twentieth century, it was discovered that the preoptic area shares various gene markers with the essentially GABAergic telencephalic subpallium, in contrast with the essentially glutamatergic PA phenotype, which led to the former’s renewed telencephalic adscription versus the latter’s alar hypothalamic adscription. We found scarce periventricular preoptic TH cells, with exception of a cell-dense TH-positive nucleus found just under the anterior commissure (the latter defines the preoptic- and THy-related roofplate); this cell group lies in the neighborhood of the intratelencephalic BST nuclear complex. We labeled it accordingly as *strial preoptic nucleus* (POSt). It seems to correspond to the dopaminergic cell group conventionally identified as ‘parvocellular anterior PA nucleus’ (pAPa; Swanson and Sawchenko, 1980), but we argue that no cell population contacting the roofplate (the anterior commissure) can belong to the PA area, that is, the alar hypothalamus, and must belong to the interposed telencephalic subpallium (hp2 preoptic area, in this case). Similarly, two constant medials (deep) hyperdorsal TH-positive cell groups with large neurons are observed above the Dpa nucleus (at the transition into the telencephalon of the Phy or hp1). These are conventionally identified as ‘magnocellular anterior PA formations.’ We likewise doubt the implied ascription of these neurons to the PA complex, considering them barely *intratelencephalic*. We accordingly have renamed them alternatively as *inner and outer magnocellular preoptic nuclei* (iPOMg; oPOMg). In this case, we think that their vicinity with the POSt nucleus, plus their topology altogether dorsal and intratelencephalic, compared with the PA complex, may justify the hypothesis that they mark an extension of the preoptic subpallial field into the hp1 prosomere (previously thought to be devoid of preoptic elements). This is a tentative proposal that has some advantages (e.g., all four subpallial domains –striatum, pallidum, diagonal area, and preoptic area- then show parallel topologies across hp2 and hp1, extending parallel to each other along the oblique septo-amygdalar axis). However, other conceptual options for ascribing these topographically hyperdorsal, thus non-hypothalamic, TH-positive cell groups have appeared recently. For instance, Morales et al. (2021) have defined recently a *Foxg1*-positive *telencephalo-opto-hypothalamic domain* (rich in *Otp* cells) that appears intercalated between the subpallium and the PA area; this might correspond to our presently postulated *extended preoptic area domain* and might contain both the POSt and the i/oPOMg formations. On the other hand, a recent study of *Sim1*-positive hypothalamic neurons that invade parts of the intratelencephalic pallial amygdala passing along the bottom of the interventricular foramen and the terminal sulcus suggested the postulate of a *hypothalamo-amygdalar corri*dor (HyA), again at the interface of subpallium and the PA area (this HyA ‘corridor’ entity, however, is seen substantially as a dorsalward deformed extension of the PA area proper; see Garcia-Calero et al., 2021). Further studies will be needed to clarify this transitional region between telencephalon (including preoptic area) and alar hypothalamus. In the meantime, our conclusion is that there exist some TH-positive cell groups that clearly lie topologically *dorsal* to the PA area proper (i.e., closer to the roofplate); these groups probably correspond to one of the newly defined subpallial or PA subdomains (extended POA; TOH; HyA).

Regarding the basal hypothalamic TH populations recognized by Puelles et al. (2012a), the classic *A13 group* was then first ascribed to the hypothalamus (rather than to the prethalamus, as most earlier publications cited above had assumed; a notable exception was Tillet, 1994). The existence of an A13 commissure or decussation was also identified (Puelles et al., 2012a; their Figures 8.25A–D). Our present analysis suggests that the compact core of this distinct nucleus is indeed strictly basal and lies in the caudal end of the RtuD domain, whereas the dorsal part of the associated shell formation seems to lie within the alar PSPa (this may be primary or, more probably, the result of secondary migratory dispersion from below).

The *dorsal tuberal group* was originally defined as lying strictly dorsal to the VM nucleus within TuD, as a distinct component of the classic A12 group (A12dt; Puelles et al., 2012a; their Figures 8.25C–D). We have now observed that this group corresponds in fact to a linear (longitudinal) band of similar cells associated to the whole TuD/RtuD domain, that is, extending through most of THy and Phy, excepting the acroterminal rostral portion and the caudal locus where the A13 group is found (see section “Results”). That this configuration was not discovered before may relate to the higher sensitivity of the present immunocytochemical protocol. Nevertheless, there is at the middle of this band a slightly more radially extensive subarea that protrudes lateralwards in a triangular shape between the VM and the DM-P nuclei. It can be distinguished from the DM-P nucleus because the latter (a RtuI formation) has a characteristic diffuse TH-positive neuropil that is absent along the TuD/RtuD area occupied by the dorsal tuberal population.

The *ventral tuberal* TH-positive cell group is found essentially along the peripheral shell portion of the DM-P and DM-T formations. TH-positive cells are most abundant at the rostral end of DM-T next to the Arc, whereas the density of such cells decreases gradientally caudalwards into DM-P. This description basically agrees with that of Puelles et al. (2012a) but introduces the precision of a topography largely coinciding with the DM shell component, essentially formed by GABAergic neurons (Puelles et al., 2012a; their Figures 8.17, 8.18, 8.20A). The DM core cell mass, contrarily glutamatergic in nature, seems devoid of TH neurons, similarly, as the largely glutamatergic ventral premamillary nucleus (VPM) that occupies a rostrolateral part of DM-T after its tangential migration from the retromamillary area (López-González et al., 2021).

Our results relative to the acroterminal *arcuate nucleus* (Arc/ArcW) corroborate the results of Puelles et al. (2012a). We noted the variable topographic relationships of arcuate TH cells with diverse peptidergic cell populations along the radial dimension. The neighboring median eminence and infundibulum show no TH cells but have a dense plexus of TH-positive terminals and fibers. Not so, however, the TH-negative acroterminal *tubero-mamillary area* (identified as Vtu in Puelles et al., 2012a), representing the rostral end of the TuV/RtuV domain, which apparently coincides at ependymal level with the hypothalamic ventricular organ (HVC), associated with the origin of histaminergic neurons; the latter disperse by short-range tangential migrations in the tubero-mamillary and mamillary/retromamillary areas (Puelles et al., 2012a; their Figures 8.28, J–Y). Few TH cells were found within the mamillary body, whereas distinct medial and lateral TH elements (RMM, RML) were identified by us at the retromamillary area. A separate small group of large radially disposed of TH neurons was detected in the caudal part of the RML, close to the rostral end of the prethalamic component of the substantia nigra compacta. This element may represent a minute *nigral population* within the retromamillary area (SNC; Figure 15B). If they are truly hypothalamic rather than diencephalic, this would convert the mesodiencephalic SNC modular continuum into a meso-diencephalo-hypothalamic complex.

### Prosomeric Topologic Interpretation of the Hypothalamic Tyrosine Hydroxylase Cell Groups

By reference to the prosomeric model of the hypothalamus, most of the studied TH populations represent longitudinally elongated dorsoventral tiers stretching more or less regularly across either the alar or basal plates of Phy and THy (prosomeres hp1, hp2), at different DV positions. In the alar plate, we have dorsalmost the hypothetically preoptic cell groups POSt and i/oPOMc, which occupy distinct hyperdorsal discontinuous positions within what could be an extended BST-related part of the preoptic area or a telencephalo-opto-hypothalamic domain (Morales et al., 2021) across hp2 and hp1. Complemented by additional periventricular elements found within the standard preoptic region, the mentioned cell populations would correspond to the classic A15 (Figures 15A,B). Underneath (ventral from) these preoptic elements there is the TH-poor Dpa subdomain of the Pa/SPa domain. This Dpa area would roughly correspond to the free space between A15 and A14 in the classic schema (Figure 15A). Ventral to that we have a variety of alar TH cell populations at various radial levels of the longitudinal Cpa (central) and Vpa (ventral) subdomains (across both THy and Phy). This includes dense periventricular elements, a variety of cells mixed with the medial subnuclei of the Pa nuclear complex (=medial or inner intermediate hypothalamic stratum), laterolateral displaced accessory elements (llC/llV = outer intermediate hypothalamic stratum), and the superficial subpial elements at the SO nucleus (jointly with the ectopic, tangentially ventrally migrated cells of the TuSbO nucleus). This radially varied system of cell groups associated with the Pa progenitor domain and nuclear complex probably should be distinguished from the SPa counterparts as *dorsal A14* (A14d; Figures 15A,B). Apart from the likewise rich SPa periventricular TH cells forming a third longitudinal alar band across THy and Phy ventrally to the Pa complex, there are relatively fewer TH neurons at the medial or inner intermediate stratum of the SPa area (these are found predominantly at the anterior hypothalamic nucleus). This less important SPa system of TH cells would form the *ventral A14* cell group (A14v; Figure 15B). Given that many embryonic neurons born at the SPa migrate tangentially into the underlying tuberal/retrotuberal basal hypothalamic domain (notably into the shell of the VM nucleus; Díaz et al., 2015), it is conceivable that some TH neurons of SPa origin may have reproduced such behavior and occupy basal locations in the adult brain. Corroboration of this possibility requires further research distinguishing theoretically alar-born from basal-born tuberal TH cells.

In general, the alar part of the acroterminal domain (including the SPa SCH nucleus) is free of TH cells in both the classic alphanumeric and prosomeric maps (Figures 15A,B). This alar Aterm represents as a whole the rostralmost transversal alar territory within the prosomeric model.

The basal hypothalamus also shows a distinct dorsoventral division of the longitudinally arranged tuberal TH cells of the classic A12 cell group into *dorsal A12* and *ventral A12* subgroups. These are aligned, respectively, with the dorsoventrally arranged TuD/RtuD and TuI/RtuI progenitor subdomains extending along THy and Phy: the corresponding TH cells appear associated (mixed) to other neuronal components of the Abas/Pbas and DM nuclei, respectively. In contrast to the uniformly poor alar acroterminal TH pattern, the related basal Aterm parts are different one from another. The AbasM Aterm formation of the TuD mantle is largely devoid of TH neurons, whereas the underlying TuI Aterm portion shows the densely TH-populated arcuate nucleus (Arc; Figure 15B), as well as the strongly innervated median eminences and hypophyseal infundibulum (Puelles et al., 2012a; present Results). However, the underlying tuberomammillary Aterm portion is devoid of TH elements similar to the underlying PM and M Aterm parts of THy.

The dorsal A12 extending along Abas/Pbas (TuD/RtuD) was thus already identified as ‘*dorsal tuberal cell grou*p’ (A12dt) by Puelles et al. (2012a); their Figure 8.25, though they did not observe the full longitudinal extent of this cell group, recognized in the present analysis apparently due to more sensitive antibody and/or protocol properties. These authors nevertheless did include the classic A13 group within the caudal PHy end of this specific longitudinal domain (A13 within Phy, caudal to Pbas; Figures 15A,B, rather than in the neighboring diencephalic prethalamus), a point we corroborated, particularly by noticing a direct relationship of A13 with the hypothalamic source of MCH neurons (see below). As far as we know, a partially hypothalamic nature of A13 was first suggested by Tillet (1994), p.211, who approximated our current interpretation by reporting that A13 neurons lie [partly] in the dorsomedial hypothalamus and [partly] in the [prethalamic] zona incerta. We think that any incertal TH elements would be alar prethalamic, and would probably correspond rather to the A11 group, though the latter was classically interpreted as tegmental (Figure 15A); the rostralmost prethalamic A11 cells, associated topographically to the zona incerta (Puelles et al., 2021), may easily be confused with those of A13. A remarkable peculiarity of the A13 group (particularly its distinct dense core portion) is that at its level there is no periventricular TH cell population, while there exists a localized decussation of TH-positive A13 fibers across the locally fused or absent ependym (Puelles et al., 2012a; their Figure 8.25B–D). In addition, the Phy RtuD progenitor domain corresponding to A13 shows in our material the apparent local origin of medial and lateral hypothalamic MCH neurons (Figures 14A–C). The latter does not extend significantly into the Pbas or Abas mantle domains. Therefore, it appears justified to maintain the distinction between A12d and A13, though their DV position is essentially the same; however, note they are distinct in terms of AP position.

**FIGURE 13:**
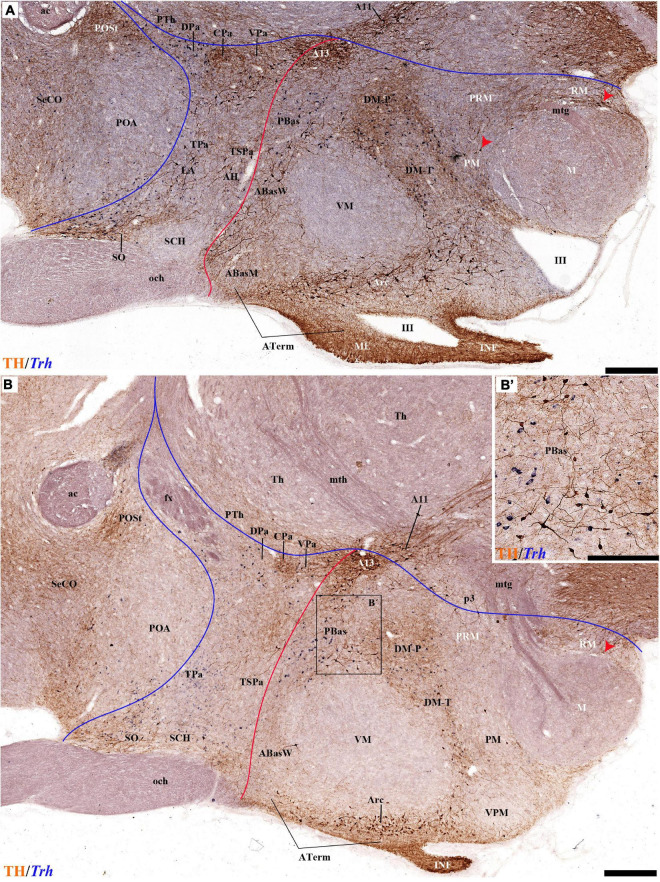
**(A,B)** TH immunohistochemistry and *Trh* ISH in two sagittal sections from an adolescent rat brain. Alar TH cells appear at the CPa, VPa, and SO nuclei; some appear at the LA and AH nuclei. *Trh* cells stand out mainly at the DPa, but also CPa and VPa. Basal TH immunoreaction is found in the A13 cell group, and the PBas, ABasW, Arc, and DM-T nuclei. Note that *Trh* cells appear in the PBas and DM-P nuclei. **(B′)** higher magnification from the boxed area in **(B)** detailing the PBas nucleus with TH immunoreactive and *Trh* positive cells. Some TH-positive cells are observed also in the PM and RM subdomains (red arrowhead). The mamillotegmental and mamillothalamic tracts are visible in (**B**; mtg; mth). For abbreviations see the list. Scale bar = 500 μm.

**FIGURE 14:**
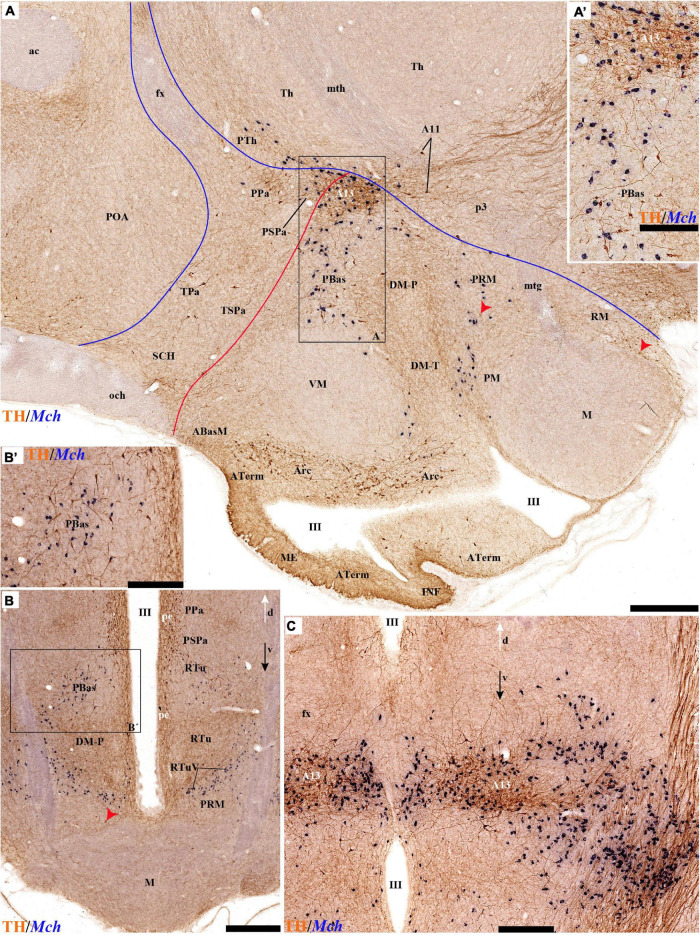
**(A)** TH immunohistochemistry and *Mch* ISH in a sagittal section of an adolescent rat brain. **(A′)** The magnified boxed region from **(A)** highlighting the A13 and PBas nuclei. **(B)** transversal section through the PHy reacted for TH immunohistochemistry and *Mch* ISH. **(B′)** Magnified region of the boxed area from **(B)**, highlighting the PBas nucleus with TH and *Mch* positive cells. **(C)** TH immunohistochemistry and *Mch* ISH in a transversal section through PHy highlighting the TH-positive A13 group, here absent at the periventricular stratum. Note numerous large-size *Mch*-positive neurons within the pe, medial, and lateral hypothalamic strata of the RTuD domain correlative with the topography of A13. For abbreviations see the list. Orienting arrows: white arrow = dorsal; black arrow = ventral. Scale bar = 500 μm.

The ventral tuberal cell group was defined as ‘*ventral tuberal ban*d’ (A12vt) by Puelles et al. (2012a). It runs basically along the periventricular and medial strata of the peduncular and terminal parts of the DM nucleus (TuI/RtuI). The TH-positive DM population is generally most abundant within the shell region of the DM nuclei at all levels (DM-T; DM-P) but changes caudalwards from one nucleus into the other in a decreasing gradient. These TH cells typically eschew populating the periventricular and medial strata of the VM nucleus (also located within TuI in late embryos and adults). This cell mass was interpreted by Puelles et al. (2012a; their Figure 8.26), as a conglomerate of diverse cell populations migrated tangentially out of the TuD, a point they demonstrated partially for its *Nkx2.2*-positive cell subpopulation, possibly related to prodynorphin neurons (*ibid*; same Figures 8.26; A-K and L-N, respectively). Interestingly, the glutamatergic TuI/RtuI populations associated with VM, DM, and VPM core nuclear parts seem completely devoid of TH cells.

**FIGURE 15:**
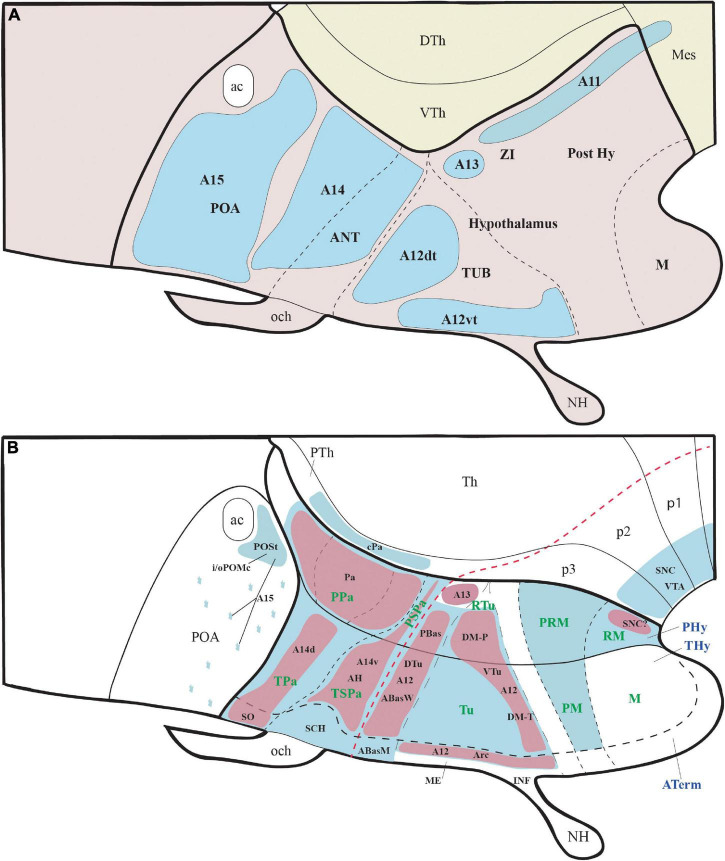
**(A,B)** schematic comparisons of the hypothalamic alphanumeric TH cell groups referred to the columnar model and the TH-positive cells groups emphasized in the present prosomeric analysis. **(A)** Relationships between the columnar anteroposterior hypothalamic regions (POA; ANT; TUB; Post Hy; M) with the standard alpha-numeric classification of local TH-positive cell groups (distinguished in pale blue). **(B)** Topological prosomeric mapping of the terminal and peduncular hypothalamic TH-positive cell populations divided into acroterminal and THy/PHy alar and basal plate territories (red dash line = alar-basal boundary). The bluish areas represent periventricular TH populations, whereas the reddish areas represent TH-positive neurons identified in the medial, lateral, or superficial strata as part of various specific nuclei (identified in the schema). For abbreviations see the list.

The hypothalamic basal region underlying the described tuberal/retrotuberal region contains in the prosomeric model other three longitudinal basal bands, the TuV/RtuV, PM/PRM, and M/RM domains (Figure 1B). These were classically held to be largely devoid of TH cells, including their respective acroterminal subdomains (Figures 15A,B). We confirmed a lack of TH cells or TH innervation comparable to that of the ME for the tuberomamillary Aterm recess, as well as along the thin TuV/RtuV territory. The latter is known conventionally as the ‘tuberomamillary band’ (i.e., it forms the longitudinal boundary between the tuberal/retrotuberal and perimamillary/periretromamillary domains in our model; Figure 1B). This thin limiting domain was identified by Puelles et al. (2012a; their Figures 8.28J-Y), as the longitudinal embryonic site where hypothalamic histaminergic neurons are produced, with local selective expression of *Arx*, *Lhx6*, and *Hdc* (histidine decarboxylase); note the postmitotic histaminergic neurons thereafter spread variously into adjacent subregions, some of which may receive dopaminergic input, allowing in principle some dopaminergic modulation of the histaminergic system at its origin. The TuV/RtuV ependym also appears developmentally related to a possible secondary DV organizer, in so far as this band of the neuroepithelium, the so-called *hypothalamic ventricular organ* (HVO; Puelles et al., 2012a; Puelles, 2017; Díaz and Puelles, 2020) expresses *Wnt8* and releases the WNT8 morphogen protein.

We found that the PM/PRM basal hypothalamic longitudinal band that surrounds the ventralmost M/RM territory shows a periventricular population of TH cells, which is absent at the corresponding Aterm area (Figure 15B).

In contrast, the underlying ventralmost M/RM longitudinal basal domain lying adjacent to the floorplate shows a fair population of TH cells, but mainly at periventricular, medial, and lateral radial levels of the RM area (periventricular, medial, and lateral RM nuclei; see comments above). The mamillary body and its Aterm subarea are largely devoid of TH neurons, though we saw some large positive cells ventrally next to the floorplate at the M/RM border.

It may be deduced that patterning phenomena acting along the hypothalamic DV dimension must be particularly relevant for explaining most of these patterns related to the differentiation of TH-positive presumptive dopaminergic neurons at different DV positions. On the other hand, AP patterning or AP attractor/repulsion signals may be required to generate the partial differential patterns displayed by the caudal Pa (cPa) elements interpreted by us as displaced into alar central prethalamus, the distinct basal peduncular A13 mass, the alar SPa AH cell population and the lack of TH cells at the SCH nucleus within the SPa Aterm area, plus the alar acroterminal SO/TuSbO and basal acroterminal Arc populations, which display distinct AP coordinates accompanied in every case by equally specific DV positions. Interestingly, the prospective locus of the infundibulum, median eminence, and Arc nucleus, which are distinguished from all other acroterminal areas by their intense TH innervation and differentiation of many small TH cells, corresponds to the rostromedian basal hypothalamic area where the endodermal prechordal plate attaches intimately during a transient early postgastrulation period up to the stage in which the adenohypophysis (Rathke’s pouch) starts to develop (Ferran et al., 2022, in press).

### Comparisons With the Alphanumeric Classification of Tyrosine Hydroxylase Cell Groups

Dahlström and Fuxe (1964) analyzed catecholamine-containing cells in the brain of rats and classified them using an alphanumeric system that recognized initially twelve cell groups (A1–12), five of which were dopaminergic (A8-A12). This list was later expanded to seventeen components (A1–A17) (Hökfelt et al., 1984). The anatomical location of dopaminergic groups A8-A15 was defined pragmatically according to their rough topography within the midbrain and diencephalon (note the preoptic area was then ascribed to the diencephalic hypothalamus, following the columnar model). The hypothalamic cell groups included A11 in the posterior hypothalamus region (Post Hy; Figure 15A), A12 in the tuberal region (TUB; Figure 15A), A14 in or near the anterior hypothalamic region (ANT; Figure 15A), and A15 in the preoptic area (POA; Figure 15A) (e.g., Chan-Palay et al., 1984; Ruggiero et al., 1984; Van den Pol et al., 1984; Björklund and Dunnett, 2007). The A13 cell group was generally described as ventral thalamic (or subthalamic; see Smeets and Reiner, 1994), though Tillet (1994) thought that part of it was placed in the dorsomedial hypothalamic area, and Puelles et al. (2012a) included it wholly in the hypothalamus. Several rodent studies reached the conclusion that hypothalamic TH neurons are presumably dopaminergic (Swanson and Hartman, 1975; Swanson et al., 1981; Chan-Palay et al., 1984; Ruggiero et al., 1984; Van den Pol et al., 1984; Ugrumov et al., 1989; Björklund and Dunnett, 2007).

However, after mapping these conventional cell groups within the prosomeric model (implying a different forebrain axis and the hypothalamus held to lie rostral to the diencephalon, plus two neuromeric hypothalamic parts -THy, Phy- and floor, basal, alar, and roof plates according to His, 1893, 1904, and a new acroterminal area; Puelles et al., 2012a; Puelles and Rubenstein, 2015; Díaz and Puelles, 2020), we excluded group A11 from the hypothalamic region because it relates to diencephalic prosomeres 1, 2 and 3 (Skagerberg and Lindvall, 1985; Marín et al., 2005; Puelles et al., 2012a,^2021^; Ozawa et al., 2017). The dopaminergic group A15 is clearly preoptic, and thus belongs to the non-evaginated telencephalon within the prosomeric model (Figure 15B; Shimogori et al., 2010; Puelles et al., 2012a). Only the conventional groups A12, A13, A14 can be considered part of the hypothalamic region as defined in the prosomeric model (Puelles et al., 2012a,^2015^).

Our analysis further reveals that the alphanumeric classification is topographically imprecise in several aspects and thus has limited utility, particularly for causal understanding.

The A14 group, which was generally vaguely described, corresponds to TH-positive cells located in the periventricular stratum of the current PA and SPa regions of the terminal and peduncular prosomeres (Puelles et al., 2012a; Ferran et al., 2015a; Puelles and Rubenstein, 2015). The standard concept of group A14 thus does not differentiate between these two molecularly and functionally distinct areas (Figure 15A, compare with **B**; note that Pa largely contains glutamatergic neurons, whereas SPa forms mainly GABAergic neurons as a result of their different molecular profiles; Shimogori et al., 2010; Puelles et al., 2012a). We accordingly propose to subdivide A14 into *A14d*, corresponding to a complex, diversely stratified set of TH cells within Pa, and *A14v*, including the less important TH populations of the underlying SPa area.

In its turn, the conventional A12 or tubero-infundibular dopaminergic group was held to include both the acroterminal arcuate TH cells (Arc; Puelles and Rubenstein, 2015) and the non-acroterminal neighboring periventricular TH neurons of the tuberal/retrotuberal hypothalamus regions (Dahlström and Fuxe, 1964; Baker et al., 1983; Lookingland and Moore, 2005). Following the prosomeric model, the so-called “mediobasal hypothalamus” notion lumps the prosomeric acroterminal tuberal, terminal tuberal, and peduncular retrotuberal hypothalamic regions that encompass distinct basal subareas such as Abas, Pbas, Arc, VM, DM-T, DM-P, each of which has differential properties (Baker et al., 1983; Puelles et al., 2012a; Puelles and Rubenstein, 2015). Even the division by Puelles et al. (2012a) of A12 into a dorsal tuberal part (A12dt) mainly related to the pe stratum of the dorsal tuberal region, and a ventral tuberal part (A12vt) defined by TH-positive cells in the Arc nucleus and in the pe stratum of the intermediate tuberal region seems now insufficient. Leaving aside the TH-poor VM nuclear complex, we propose to distinguish as probably causally independent the *acroterminal Arc group* from the *dorsal and ventral tuberal groups*, associated, respectively, to the TuD/RtuD domain (A12dtu) or to the TuI/RtuI domain occupied largely by the DM nucleus (A12vtu; DM-T + DM-P; Figure 15B). Each of these components probably has a different AP or DV causal explanation, according to their different topologic positions, and it is possible that their respective periventricular TH cells also display corresponding differential properties.

The dopaminergic A13 group was sometimes defined as incerto-hypothalamic (IH) and described as located in the rostral portion of the zona incerta (Chan-Palay et al., 1984; Van den Pol et al., 1984; Puelles and Medina, 1994). The latter is now classified as a ventral alar part of the prethalamus within the prosomeric model (the prethalamic alar subcentral complex; Puelles et al., 2021). There are indeed several TH neurons just underneath this diencephalic area, extending back across a similar liminar alar thalamic band into the pretectum. However, we identify this longitudinal diencephalic band as corresponding to the conventional A11 cell group. We follow instead Puelles et al. (2012a) in identifying the A13 with a much denser core of TH neurons found mainly in the medial stratum of the caudal end of the RtuD domain of Phy (surrounded by a sparser shell population). Our demonstration that this cell group coincides topographically with hypothalamic MCH neurons of the medial and lateral strata of the Phy corroborates this interpretation. Baker et al. (1983) thought that the ‘mediodorsal’ hypothalamus contains TH-positive neurons from groups A13 and A14. Both groups are strictly outside the DM complex, which is populated instead by its own A12vtu cell group, usually present in the shell portion that surrounds the dense core portion. The doubtful description of these authors apparently confused the peduncular alar PA nucleus (Pa derivative) with the peduncular basal A13 and underlying DM nucleus (Figure 15B).

A, similarly, imprecise notion is that of the periventricular nucleus of Chan-Palay et al. (1984). According to these authors this population lying close to the third ventricle extends uninterruptedly from the PA and anterior hypothalamic nuclei (alar hypothalamus) to the suprachiasmatic, arcuate, and dorsomedial nuclei (basal hypothalamus), being absent only at the ventromedial nucleus (Chan-Palay et al., 1984). A causal explanation of its patterning and origin as a single entity would be difficult; we think that a periventricular position is not a sufficiently precise character for defining and naming a cell group (i.e., most parts of the brain have periventricular neurons) and use of such a concept merely hampers our analysis of more specific entities. It seems thus advisable to separate at least the alar and basal components (strictly different molecular environments), and our functional understanding might profit as well from distinguishing likewise DV portions ascribed developmentally to alar Pa and SPa and dorsal versus intermediate basal Tu/Rtu parts. Some functional and hodological studies suggest that periventricular cells of the hypothalamus do not function as a unit (Goudreau et al., 1995; Zhang and van den Pol, 2016). For instance, a group of paraventriculo-hypophysial neurons of our A14d cell group includes a subset of dopaminergic cells that project to the intermediate lobe of the posterior pituitary (Goudreau et al., 1995). Future studies should clarify the potential association of each distinct population of periventricular TH cells with differential connections and functions.

## Data Availability Statement

The original contributions presented in the study are included in the article/Supplementary Material, further inquiries can be directed to the corresponding author.

## Ethics Statement

The animal study was reviewed and approved by Comité Ético de Experimentación Animal (CEEA), Universidad de Murcia.

## Author Contributions

MB, DG, MM-M, and JF contributed to design. MB, DG, MM-M, AT, YK, RB, AB, BR, and JF contributed to experiment/data collection. MB, DG, MM-M, LP, and JF contributed to analysis. LP and JF contributed to writing-original draft. MB, DG, MM-M, AT, YK, RB, AB, BR, LP, and JF contributed to writing-review and editing. JF contributed to funding acquisition and project administration. All authors contributed to the article and approved the submitted version.

## Conflict of Interest

The authors declare that the research was conducted in the absence of any commercial or financial relationships that could be construed as a potential conflict of interest.

## Publisher’s Note

All claims expressed in this article are solely those of the authors and do not necessarily represent those of their affiliated organizations, or those of the publisher, the editors and the reviewers. Any product that may be evaluated in this article, or claim that may be made by its manufacturer, is not guaranteed or endorsed by the publisher.

## Glossary

A11: group of A11 catecholaminergic cells
A12dt: dorsal tuberal part of group A12
A12it: intermediate tuberal part of group A12
A12vt: ventral tuberal part of group A12
A13: group of A13 catecholaminergic cells
A14d: dorsal part of group A14
A14v: ventral part of group A14
AADC: L-aromatic acid decarboxylase
ABas: anterobasal nucleus
ABasM: median anterobasal area
AbasW: wing of the anterobasal area
ac: anterior commissure
Agrp: agouti-related peptide
AH: anterior hypothalamic nucleus
AHP: anterior hypothalamic nucleus peduncular
ANT: anterior hypothalamic nucleus terminal
ap: alar plate
AP: antero-posterior
Arc: arcuate nucleus
ArcM: median arcuate nucleus
ArcW: wing of arcuate nucleus
ATerm: acroterminal region
Avp: arginine vasopressin
bp: basal plate
BST: bed nucleus of the stria terminalis
c: caudal
CA: catecholamines
Cart, cocaine and amphetamine: regulated transcript
Cb: cerebellum
cCPA: caudal central paraventricular nucleus
cDPA: caudal dorsal paraventricular nucleus
cPa: caudal paraventricular área
CPa: central paraventricular nucleus
Crh: corticotropin releasing Hormone
cVPA: caudal ventral paraventricular nucleus
Cx: cortex
d: dorsal
D: dorsal tuberal/retrotuberal region
DA: dopamine
DB: diagonal band
DBH: dopamine- β -hydroxylase
DM: dorsomedial nucleus
DM-P: peduncular dorsomedial nucleus
DM-T: terminal dorsomedial nucleus
DPa: dorsal paraventricular nucleus
DTh: dorsal thalamus
DTu: Dorsal tuberal region
DV: dorso-ventral
eTPA, external cell: terminal paraventricular area
fp: floor plate
fx: fornix
HB: hindbrain
HDB: hypothalamo-diencephalic boundary
hp1: hypothalamic prosomere 1
hp2: hypothalamic prosomere 2
HVO: hypothalamic ventricular organ
hycsm: hypothalamic caudal stria medullaris tract
I: intermediate tuberal/retrotuberal region
III: third ventricle
iC: inner central part of the paraventricular nucleus
IH: incertohypothalamic
IHB: intrahypothalamic boundary
INF: infundibulum
iPOMc: inner preoptic magnocellular nucleus
ITPa: intermediate terminal paraventricular cells
ITSPa: intermediate terminal subparaventricular cells
ITU: intermediate tuberal region
iV: inner ventral part of the paraventricular nucleus
LA: lateral anterior hypothalamic nucleus
llC: lateral central part of the paraventricular nucleus
llV: lateral lateral ventral part of the paraventricular nucleus
M: mamillary region
MB: midbrain
Mch: melanin concentrating hormone
mD: medial stratum from the dorsal paraventricular area
ME: median eminence
mfb: medial forebrain bundle
mh: medial hypothalamic stratum
mlV: medial lateral ventral part of the paraventricular nucleus
mPOSt: medial stratum of preopto-strial nucleus
mtg: mamillo-tegmental tract
mth: mamillo-thalamic tract
NH: neurohypophysis
Npy: neuropeptide Y
ns/mc: nigrostriatal and mesocortical tracts
OB: olfactory bulb
oC: outer central part of the paraventricular nucleus
och: optic chiasma
on: optic nerve
oPOMc: outer preoptic magnocellular nucleus
ot: optic tract
oV: outer ventral part of the paraventricular nucleus
Oxt: oxytocin/neurophysin I
p1: prosomere 1
p2: prosomere 2
p3: prosomere 3
Pa: paraventricular area
PBas: peduncular posterobasal area
PBS: phosphate-buffed saline
PCPa: peduncular central paraventricular progenitor subregion
PDPa: peduncular dorsal paraventricular progenitor subregion
pe: periventricular stratum
peCPa: periventricular central paraventricular parvocellular partition
ped: peduncle
peDPa: periventricular dorsal paraventricular parvocellular partition
pePOSt: periventricular preopto-strial partition
peVPa: periventricular ventral paraventricular parvocellular partition
PHy: peduncular hypothalamus
PInPFx: Pomc, proopiomelanocortin; preincertal perifornical cell group
PM: perimamillary región
PNMT: phenylethanolamine-N-methyltransferase
POA: preoptic area
POce: preoptic central region
POSt: preopto-strial nucleus
Post Hy: posterior hypothalamus
PPa: peduncular prosomere paraventricular area
PRM: periretromamillary region
PSPa: peduncular subparaventricular area
PT: pretectum
PTh: prethalamus
PVPa: peduncular ventral paraventricular progenitor subregion
r: rostral
RLi: rostral liminar area
RM: retromamillary region
RML: lateral retromamillary nucleus
RMM, medial retromamillary nucleusrp: roof plate
rt: retroflex tract
Rt: reticular nucleus
RTu: retrotuberal area
RTuD: retrotuberal dorsal hypothalamic area
RTuI: retrotuberal intermediate hypothalamic area
RTuV: retrotuberal ventral hypothalamic area
SC: spinal cord
SCH: suprachiasmatic nucleus
SD: sprague Dawley
Se: septum
SeCo: septocommisural region
smed: stria medullaris
SNC: substantia nigra compacta
SO: supraoptic nucleus
SPa, Sst,: somatostatin; subparaventricular area
TCI: tuber cinereum
Tel: telencephalon
Th: thalamus
Th: tyrosine hidroxilase
TH: Tyrosine Hydroxylase
THy: terminal hypothalamus
TPa: terminal paraventricular area
Trh: thyrotropin releasing hormone
TSPa: terminal subparaventricular area
Tu: tuberal hypothalamic área
TUB: tuberal region
TuD: tuberal dorsal hypothalamic area
TuI: tuberal intermediate hypothalamic area
TuSbO: tuberal suboptic nucleus
TuV: tuberal ventral hypothalamic area
v: ventral
V: ventral
VM: ventromedial nucleus
VMs, ventromedial nucleus: superificial
VPa: ventral paraventricular nucleus
VPM: ventral perimamillary nucleus
VTA: ventral tegmental area
VTh: ventral thalamus
VTu: ventral tuberal region
ZI: zona incerta.
