# Supplementary material for: Prosomeric Hypothalamic Distribution of Tyrosine Hydroxylase Positive Cells in Adolescent Rats
Source: Front Neuroanat. 2022 May 6;16:868345. doi: 10.3389/fnana.2022.868345 (PMC9121318; doi:10.3389/fnana.2022.868345)
Supplement: Supplementary file 1 [file Data_Sheet_1.zip › SMaterial04.pdf]

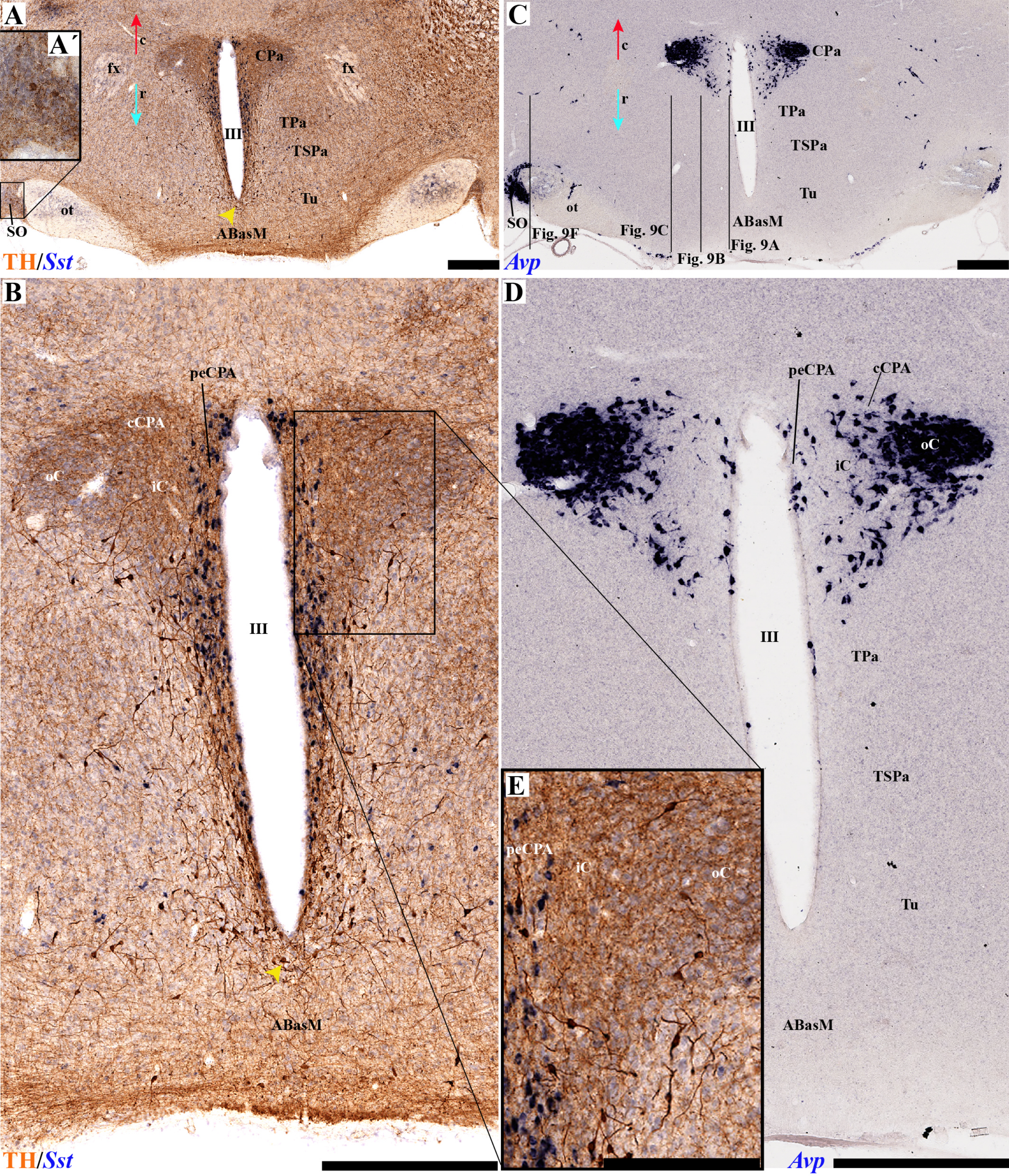

A
A´
c
r
fx
CPa
fx
TPa
III
TSPa
Tu
ot
SO
ABasM
TH/Sst
C
c
r
CPa
TPa
III
TSPa
Tu
ot
SO
ABasM
Fig. 9F
Fig. 9C
Fig. 9B
Fig. 9A
Avp
B
peCPA
cCPA
oC
iC
III
ABasM
TH/Sst
D
peCPA
cCPA
oC
iC
III
TPa
TSPa
Tu
ABasM
Avp
E
peCPA
iC
oC

Supplementary material 04: (A-E) TH immunohistochemistry combined with *Sst* ISH in a horizontal section through the alar paraventricular area (CPa) of an adolescent rat brain. Both TH immunoreaction and *Sst* ISH reaction characterize the periventricular stratum of the CPa (**A,B**; box in **B** at higher magnification in **E**). Note differential ISH mapping of *Avp* signal in an adjacent section **(C-D)**. The section planes of Figs.**9A,B,C,F** are indicated at the bottom. For abbreviations see the list. Orientation arrows: red arrow = caudal; blue arrow = rostral. Scale bar = 500 µm.
