# Supplementary material for: Prosomeric Hypothalamic Distribution of Tyrosine Hydroxylase Positive Cells in Adolescent Rats
Source: Front Neuroanat. 2022 May 6;16:868345. doi: 10.3389/fnana.2022.868345 (PMC9121318; doi:10.3389/fnana.2022.868345)
Supplement: Supplementary file 1 [file Data_Sheet_1.zip › SMaterial05.pdf]

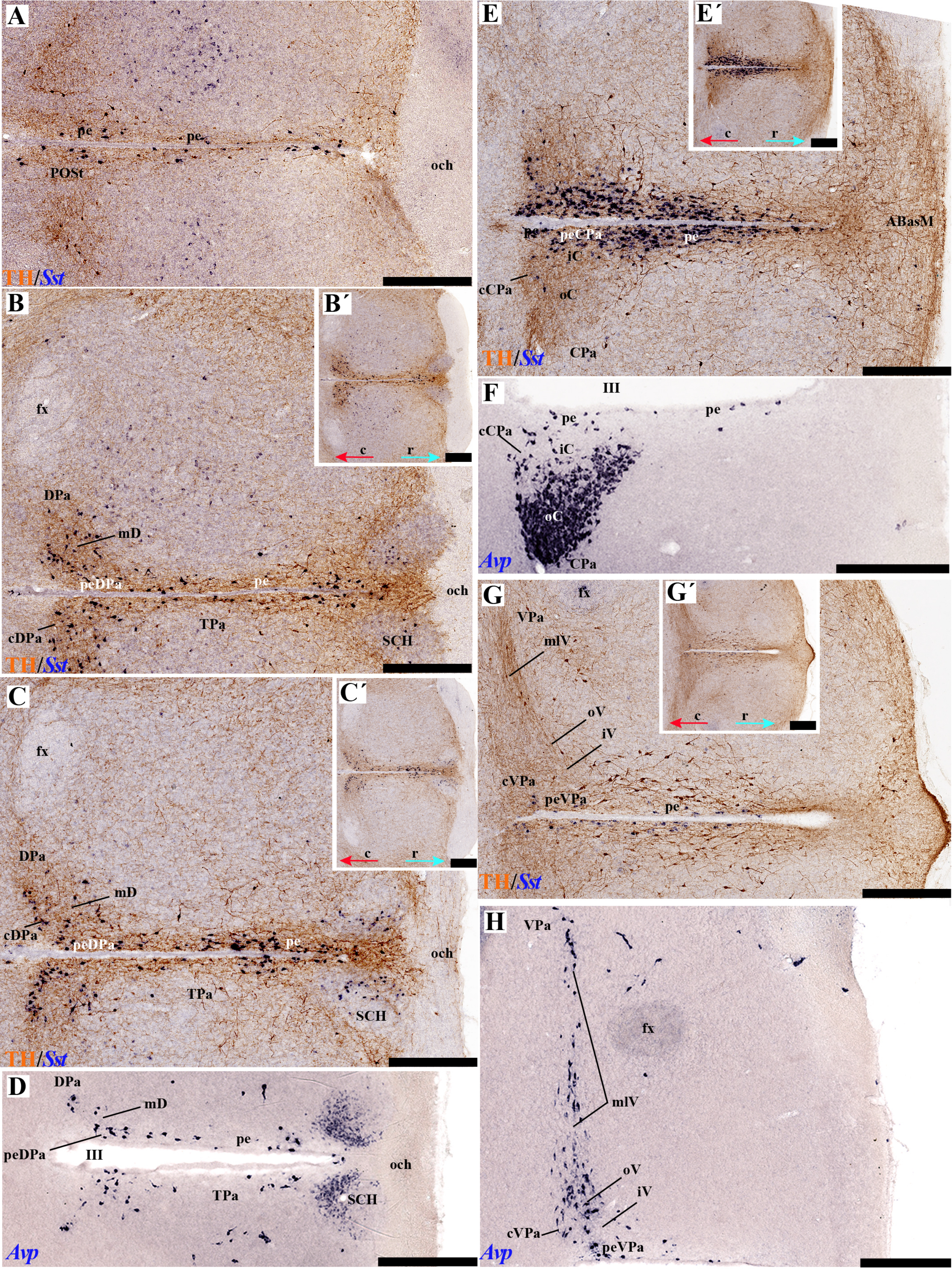

A
pe
pe
POSt
och
TH/Sst
B
B′
c
r
fx
DPa
mD
peDPa
pe
och
cDPa
TPa
SCH
TH/Sst
C
C′
c
r
fx
DPa
mD
cDPa
peDPa
pe
och
TPa
SCH
TH/Sst
D
DPa
mD
pe
peDPa
III
och
TPa
SCH
Avp
E
E′
c
r
ABasM
peCPa
pe
iC
cCPa
oC
CPa
TH/Sst
F
III
pe
pe
cCPa
iC
oC
CPa
Avp
G
G′
fx
VPa
mlV
c
r
oV
iV
cVPa
peVPa
pe
TH/Sst
H
VPa
fx
mlV
oV
iV
cVPa
peVPa
Avp

Supplementary material 05: (A-H) TH immunohistochemistry combined with *Sst* ISH in selected horizontal sections through the hypothalamic alar plate of an adolescent rat. Selected *Avp* ISH reacted sections were included for comparison. **(A)** TH and *Sst*-positive cells are observed in the preoptic POSt nucleus. **(B,B´,C,C´,D)** TH, *Sst* and *Avp* reactive cells are present in the DPa, as well as in the TPa pe stratum . Note that *Sst* and *Avp* signal appears in the shell of the SCH nucleus. **(E,E´,F)** TH, *Sst* and *Avp* signal in the CPa. Note high expression of *Sst* in the pe stratum. **(G,G´,H)** Distribution of TH immunoreactive and *Avp* positive cells in the VPa; periventricular *Sst* cells are relatively less numerous within VPa. For abbreviations see the list. Orientation arrows: red arrow = caudal; blue arrow = rostral. Scale bar = 500 µm.
