# Supplementary material for: Prosomeric Hypothalamic Distribution of Tyrosine Hydroxylase Positive Cells in Adolescent Rats
Source: Front Neuroanat. 2022 May 6;16:868345. doi: 10.3389/fnana.2022.868345 (PMC9121318; doi:10.3389/fnana.2022.868345)
Supplement: Supplementary file 1 [file Data_Sheet_1.zip › SMaterial06.pdf]

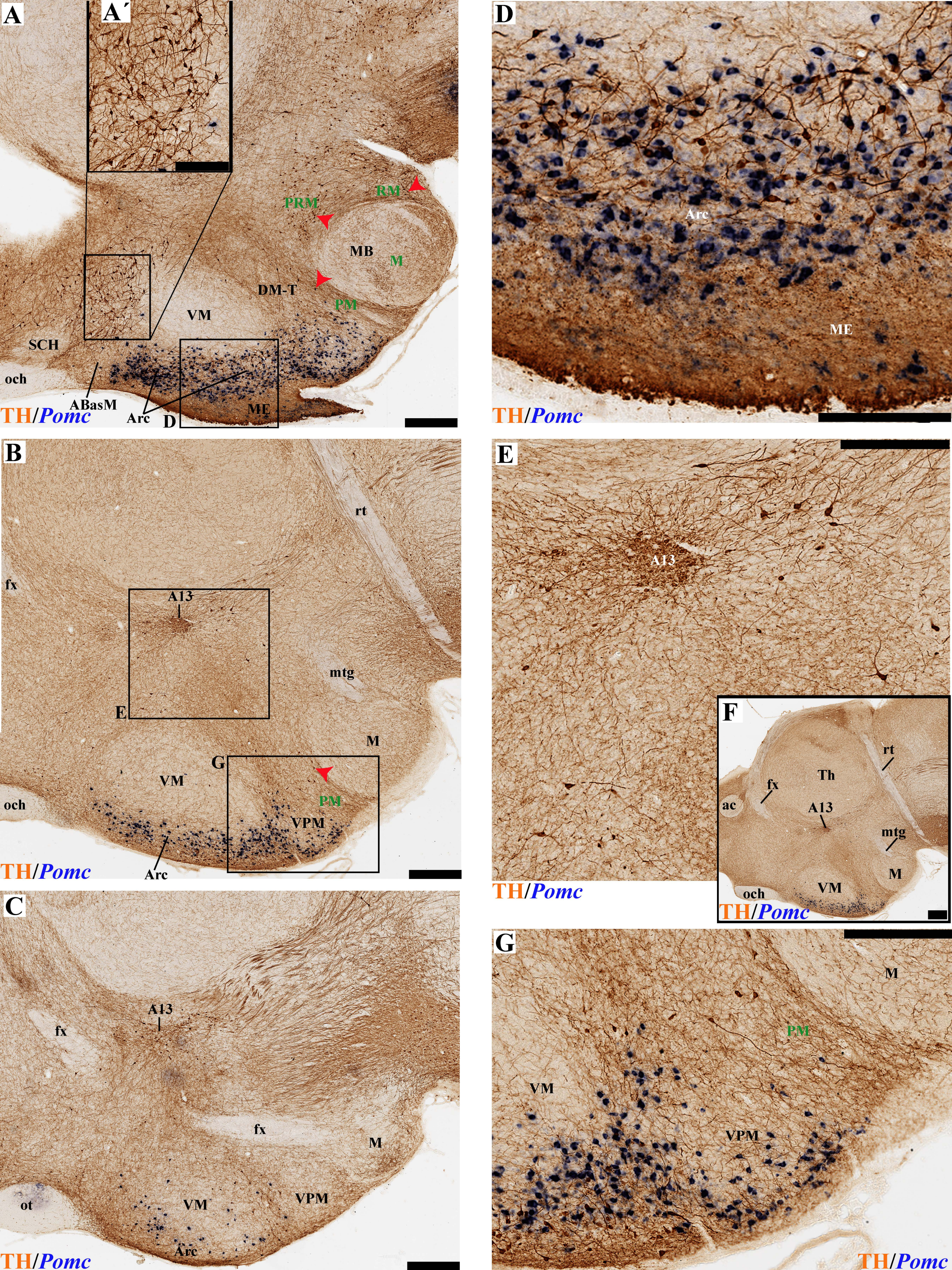

A
A′
PRM
RM
MB
M
DM-T
PM
VM
SCH
och
ABasM
Arc
D
ME
TH/Pomc
D
Arc
ME
TH/Pomc
B
rt
fx
A13
mtg
E
M
G
VM
PM
VPM
och
Arc
TH/Pomc
E
A13
TH/Pomc
F
rt
fx
Th
ac
A13
mtg
M
och
VM
TH/Pomc
C
A13
fx
fx
M
ot
VM
VPM
Arc
TH/Pomc
G
M
PM
VM
VPM
TH/Pomc

Supplementary material 06: (A-C) *Pomc* ISH and TH immunoreaction in three selected sagittal sections of an adolescent rat brain. TH is observed in the pe stratum of the Pa and SPa domains (**A'** shows a higher magnification of the pe stratum). *Pomc* positive cells are detected in the acroterminal Arc nucleus, mixed with TH immunoreactive cells. Some TH positive cells are observed in the PM and RM areas (red arrowheads). **(D)** Higher magnification of the Arc and medial eminence from (A) with *Pomc*- and TH-positive neurons. **(E)** Magnified box from **(B)** highlighting the A13 TH positive group. **(F)** Low magnification view of **(B)** showing TH-positive A13 and *Pomc* cells. **(G)** Higher magnification of a boxed area in **(B)** highlighting TH and *Pomc* expression in the Arc and VPM nuclei. See section planes in SM**7B**. For abbreviations see the list. Scale bar = 500 µm.
