# Supplementary material for: Prosomeric Hypothalamic Distribution of Tyrosine Hydroxylase Positive Cells in Adolescent Rats
Source: Front Neuroanat. 2022 May 6;16:868345. doi: 10.3389/fnana.2022.868345 (PMC9121318; doi:10.3389/fnana.2022.868345)
Supplement: Supplementary file 1 [file Data_Sheet_1.zip › SMaterial07.pdf]

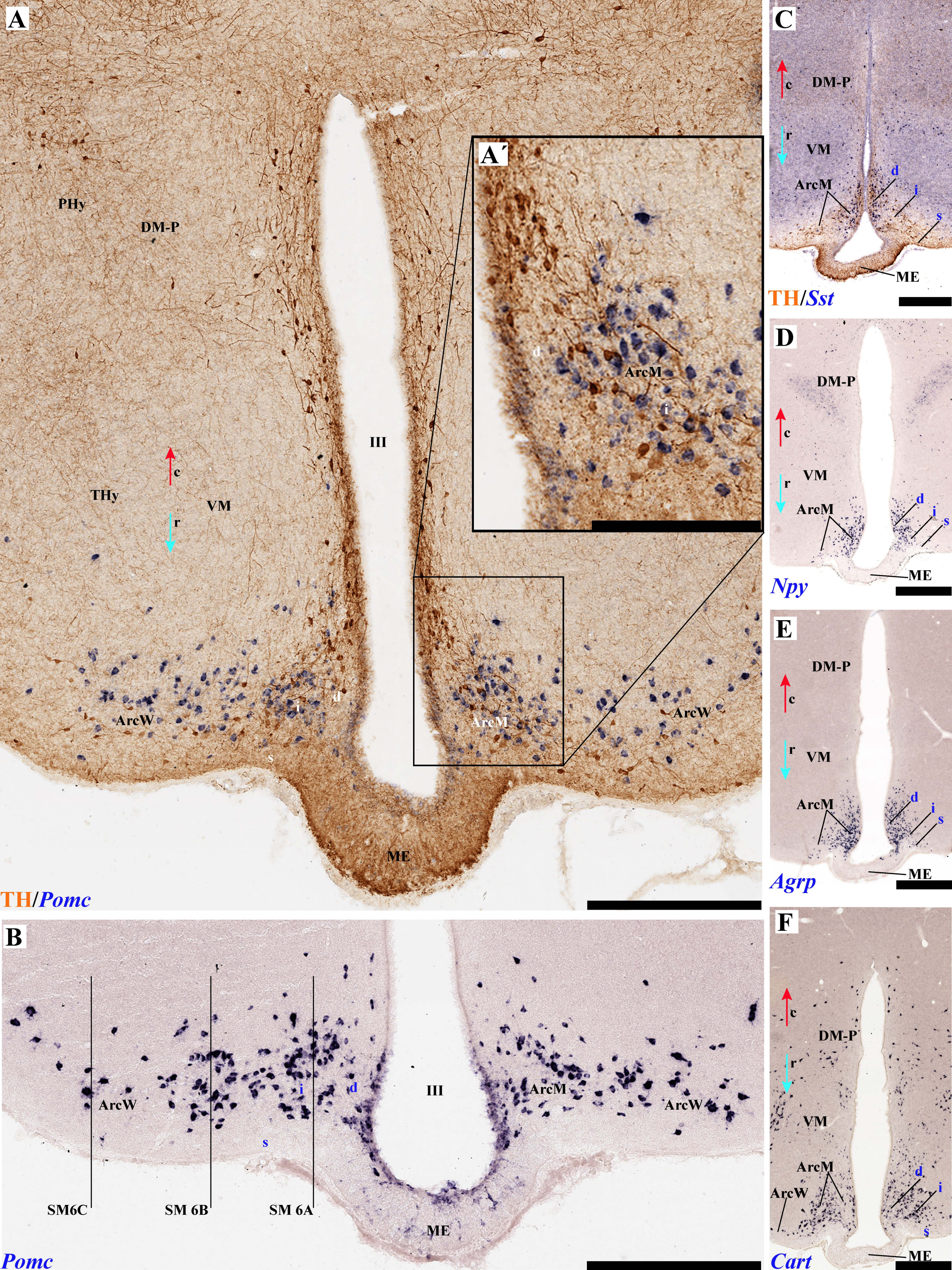

A
PHy
DM-P
THy
c
r
VM
III
ArcW
i
d
s
ArcM
ME
TH/Pomc
A´
d
ArcM
i
B
ArcW
i
d
III
ArcM
ArcW
s
SM6C
SM 6B
SM 6A
ME
Pomc
C
c
DM-P
r
VM
ArcM
d
i
s
ME
TH/Sst
D
DM-P
c
r
VM
ArcM
d
i
s
ME
Npy
E
DM-P
c
r
VM
ArcM
d
i
s
ME
Agrp
F
c
DM-P
r
VM
ArcM
ArcW
d
i
s
ME
Cart

Supplementary material 07: **(A)** TH immunohistochemistry and *Pomc* ISH reaction in a horizontal section through hypothalamic basal plate domains. Both cell types appear at the deep and intermediate strata (d,i) of the Arc nucleus. The inset A' shows a high magnification of the Arc nucleus. **(B)** adjacent section displaying *Pomc* signal distributed in the ArcM and ArcW portions. **(C-F)** more or less stratified Arc cells expressing *Sst* **(C)**, *Npy* **(D)**, *Agrp* **(E)**, and *Cart* **(F)**. The section planes of Figs.**SM6A**,**B,C,F** are indicated in **B**. For abbreviations see the list. Orientation arrows: red arrow = caudal; blue arrow = rostral. Scale bar = 500 µm.
