# Supplementary material for: Prosomeric Hypothalamic Distribution of Tyrosine Hydroxylase Positive Cells in Adolescent Rats
Source: Front Neuroanat. 2022 May 6;16:868345. doi: 10.3389/fnana.2022.868345 (PMC9121318; doi:10.3389/fnana.2022.868345)
Supplement: Supplementary file 1 [file Data_Sheet_1.zip › SMaterial08.pdf]

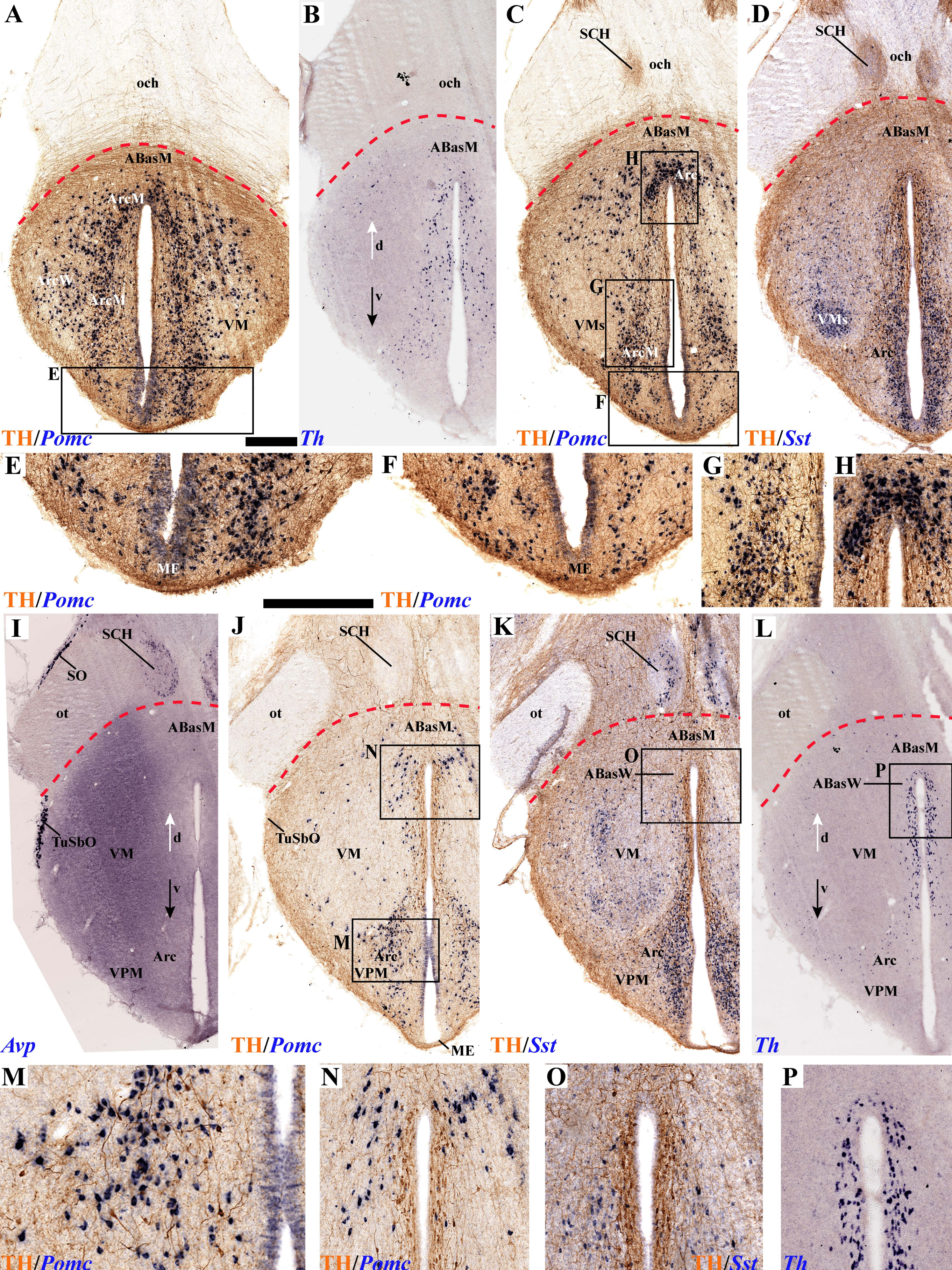

A
och
ABasM
ArcM
ArcW
ArcM
VM
E
TH/Pomc
B
och
ABasM
d
v
Th
C
SCH
och
ABasM
H
Arc
G
VMs
ArcM
F
TH/Pomc
D
SCH
och
ABasM
VMs
Arc
TH/Sst
E
ME
TH/Pomc
F
ME
TH/Pomc
G
H
I
SCH
SO
ot
ABasM
TuSbO
VM
d
v
Arc
VPM
Avp
J
SCH
ot
ABasM
N
TuSbO
VM
M
Arc
VPM
ME
TH/Pomc
K
SCH
ot
ABasM
O
ABasW
VM
Arc
VPM
TH/Sst
L
ot
ABasM
ABasW
P
d
VM
v
Arc
VPM
Th
M
TH/Pomc
N
TH/Pomc
O
TH/Sst
P
Th

Supplementary material 08: (A-D, **I-L)** Series of eight transversal sections through the ATerm and THy hypothalamic domains comparing TH/*Pomc*, *Th*, TH/*Sst* and *Avp* signals, ordered rostrocaudally, with correlative higher magnification details (**E-H**, **M-P**). **(A,C,J)** combine TH immunoreaction with *Pomc* ISH, illustrating their respective expression in the deep (d) and intermediate (i) strata of the Arc nucleus (**E-H** and **M,N** are higher magnification details from A,C and J). **(B,L)** *Th* ISH signal observed in the pe stratum of the Arc nucleus (**P** is a higher magnification of **L**). (**D**, **K**) Combined expression of TH and *Sst* ISH in the pe stratum of the Arc nucleus (**O** is a higher magnification from **K**); note the absence of *Sst* expression in the pe stratum of the PBas nucleus in **K**, and labelling in the superficial VM subnucleus, VMs, an area devoid of *Pomc* [**C**,**D]**). (**I**) note *Avp* ISH signal at the SCH shell, and the SO and TuSbO nuclei. For abbreviations see the list. Orienting arrows: white arrow = dorsal Scale bar = 500 µm.
