# Supplementary material for: Prosomeric Hypothalamic Distribution of Tyrosine Hydroxylase Positive Cells in Adolescent Rats
Source: Front Neuroanat. 2022 May 6;16:868345. doi: 10.3389/fnana.2022.868345 (PMC9121318; doi:10.3389/fnana.2022.868345)
Supplement: Supplementary file 1 [file Data_Sheet_1.zip › SMaterial09.pdf]

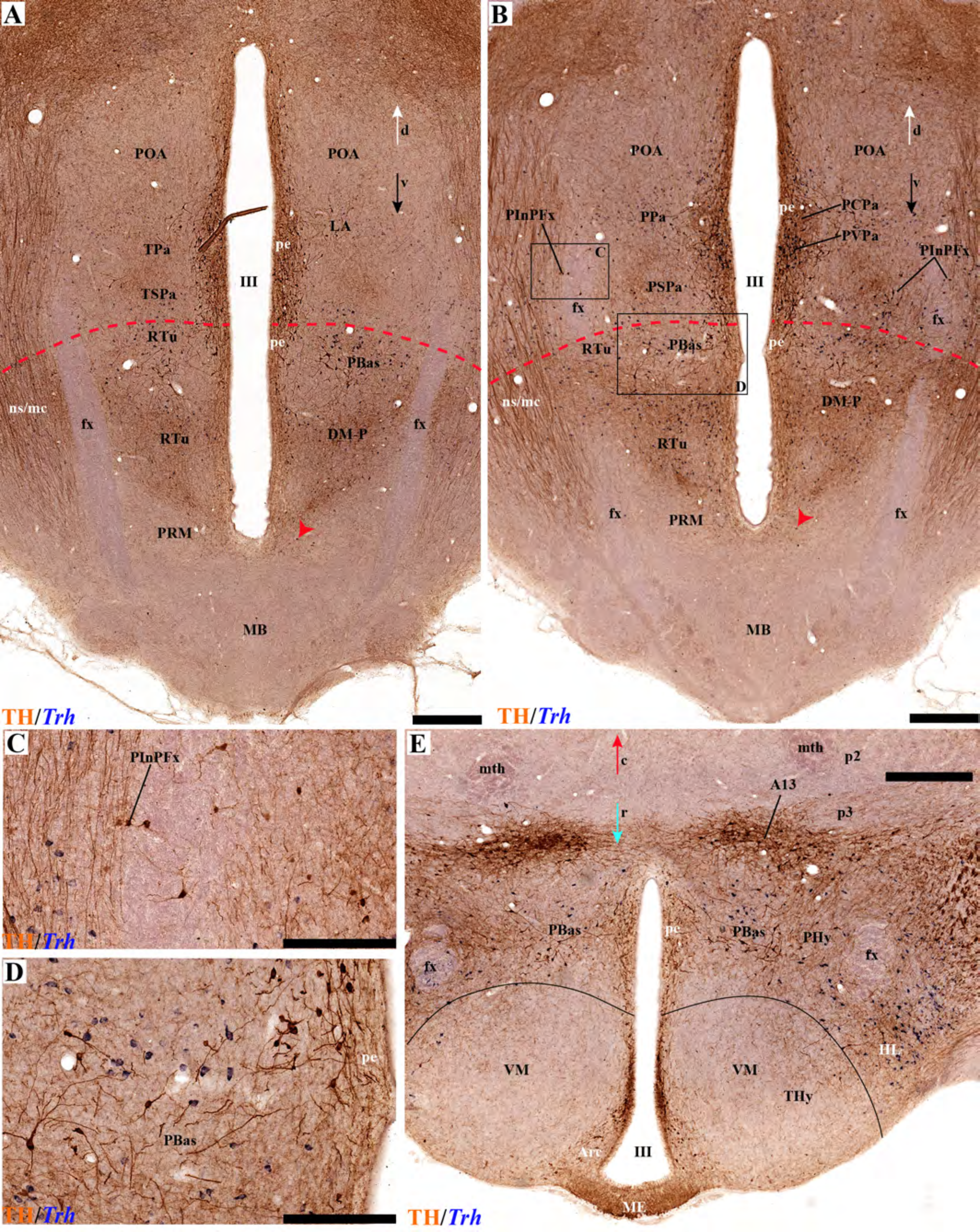

A
B
C
D
E
POA
POA
d
v
LA
TPa
TSPa
III
pe
RTu
PBas
ns/mc
fx
DM-P
PRM
MB
PInPFx
PPa
PCPa
PVPa
PSPa
TH/Trh
mth
p2
p3
c
r
A13
PHy
VM
THy
HL
Arc
ME

Supplementary material 09: (A-B) two transversal sections comparing TH immunohistochemistry with *Trh* ISH at levels through the retroflex and nigrostriatal tracts (fx; ns/mc). Note the TH/*Trh*-positive alar and basal periventricular stratum, as well as TH/*Trh* elements at the PBas and DM-P nuclei, and TH cells forming a preincertal perifornical cell group (PInPFx). Some TH cells appear at the PRM area (red arrowheads in **A,B**). The boxed areas in (**B**) are shown at higher magnification in (**C,D**). (**E**) horizontal section through the A13 cell group (PHy), and the VM nucleus and the median eminence (ME; THy), showing local TH/*Trh* positive cells, also in the superficial lateral hypothalamus (HL). For abbreviations see the list. Orienting arrows: white arrow = dorsal; black arrow = ventral; red arrow = caudal; blue arrow = rostral. Scale bar = 500 µm.
