# Supplementary material for: Prosomeric Hypothalamic Distribution of Tyrosine Hydroxylase Positive Cells in Adolescent Rats
Source: Front Neuroanat. 2022 May 6;16:868345. doi: 10.3389/fnana.2022.868345 (PMC9121318; doi:10.3389/fnana.2022.868345)
Supplement: Supplementary file 1 [file Data_Sheet_1.zip › SMaterial01.pdf]

Table 1. Primers

Gene	Forward	Reverse
Agrp	5´-TAGCTACAGGAAGTAGTCACG-3´	5´-GTGCTACTGCCGCTTCTTCAA -3´
Cart	5´-CTGCTACCTTTGCTGGGTGC-3´	5´-TGAAGCAGCAGGGAAAGAGC-3´
Mch	5´-AACAGGATGGCGAAGATGAG-3´	5´-ACATGCTCAGGTGTATGCT-3´
Npy	5´-CTCACAGAGGCACCCAGAG-3´	5´-CCATTTCCCTTGTTGTTGTTG-3´
Oxt	5´-CGACGGTGGATCTCGGACTG-3´	5´-GCAGCCCTCTTGCCGCCCAG-3´
Pomc	5´-GGAGCAGTGACTAAGAGAGG-3´	5´-CCATCATCAAGAACGCGCAC-3´
Sst	5´-CTGAAGGAGACGCTACTGGAG-3´	5´-AGACCCTGCGTTAGAAATTGAG-3´
Th	5´-GATTGCAGAGATTGCCTTCC-3´	5´-AGTGCCATTAGCTAAATGCATAGGG -3´
Trh	5´-CTGCCTTAGACTCCTGGATC-3´	5´-ATAGCTTCAGCGCATCCTCC-3´
