# Supplementary material for: Prosomeric Hypothalamic Distribution of Tyrosine Hydroxylase Positive Cells in Adolescent Rats
Source: Front Neuroanat. 2022 May 6;16:868345. doi: 10.3389/fnana.2022.868345 (PMC9121318; doi:10.3389/fnana.2022.868345)
Supplement: Supplementary file 1 [file Data_Sheet_1.zip › SMaterial02.pdf]

Table 2. List of fragment of genes used and their main characteristics.

Gen	Species	NCBI number/size/position	Laboratory/References
Agrp	*Rattus novergicus*	NM_033650.1/436bp/101-536	Present results
Avp	*Rattus novergicus*	NM_01692/495bp/17–511	Toval et al. 2020
Cart	*Rattus novergicus*	NM_017110.1/475bp/74-548	Present results
Crh	*Rattus novergicus*	NM_031019.1/927bp/141–1068	Toval et al. 2020
Mch	*Rattus novergicus*	NM_012625.1/470bp/51-520	Present results
Npy	*Rattus novergicus*	NM_012614/492 bp/12-504	Present results
Oxt	*Rattus novergicus*	X12792.1/130bp/2499-2628	Present results
Pomc	*Rattus novergicus*	NM_139326.2/776bp/39-814	Present results
Sst	*Rattus novergicus*	NM_012659.2/563bp/1-562	Present results
Th	*Rattus novergicus*	NM_012740.3/890bp/628-1518	Present results
Trh	*Rattus novergicus*	XM_006236899.2/931bp/766-1696	Present results
